# Trabecular bone patterning in the hominoid distal femur

**DOI:** 10.7717/peerj.5156

**Published:** 2018-07-05

**Authors:** Leoni Georgiou, Tracy L. Kivell, Dieter H. Pahr, Matthew M. Skinner

**Affiliations:** 1 Skeletal Biology Research Centre, School of Anthropology and Conservation, University of Kent at Canterbury, Canterbury, Kent, UK; 2 Department of Human Evolution, Max Planck Institute for Evolutionary Anthropology, Leipzig, Germany; 3 Institute for Lightweight Design and Structural Biomechanics, Vienna University of Technology, Vienna, Austria; 4 Department of Anatomy and Biomechanics, Karl Landsteiner Private University of Health Sciences, Krems an der Donau, Austria

**Keywords:** Trabecular bone, Functional morphology, Locomotion, Hominoid

## Abstract

**Background:**

In addition to external bone shape and cortical bone thickness and distribution, the distribution and orientation of internal trabecular bone across individuals and species has yielded important functional information on how bone adapts in response to load. In particular, trabecular bone analysis has played a key role in studies of human and nonhuman primate locomotion and has shown that species with different locomotor repertoires display distinct trabecular architecture in various regions of the skeleton. In this study, we analyse trabecular structure throughout the distal femur of extant hominoids and test for differences due to locomotor loading regime.

**Methods:**

Micro-computed tomography scans of *Homo sapiens* (*n* = 11), *Pan troglodytes* (*n* = 18), *Gorilla gorilla* (*n* = 14) and *Pongo* sp. (*n* = 7) were used to investigate trabecular structure throughout the distal epiphysis of the femur. We predicted that bone volume fraction (BV/TV) in the medial and lateral condyles in *Homo* would be distally concentrated and more anisotropic due to a habitual extended knee posture at the point of peak ground reaction force during bipedal locomotion, whereas great apes would show more posteriorly concentrated BV/TV and greater isotropy due to a flexed knee posture and more variable hindlimb use during locomotion.

**Results:**

Results indicate some significant differences between taxa, with the most prominent being higher BV/TV in the posterosuperior region of the condyles in *Pan* and higher BV/TV and anisotropy in the posteroinferior region in *Homo*. Furthermore, trabecular number, spacing and thickness differ significantly, mainly separating *Gorilla* from the other apes.

**Discussion:**

The trabecular architecture of the distal femur holds a functional signal linked to habitual behaviour; however, there was more similarity across taxa and greater intraspecific variability than expected. Specifically, there was a large degree of overlap in trabecular structure across the sample, and *Homo* was not as distinct as predicted. Nonetheless, this study offers a comparative sample of trabecular structure in the hominoid distal femur and can contribute to future studies of locomotion in extinct taxa.

## Introduction

Extant great apes are often used as models to help reconstruct the origin and evolution of bipedality, and to help interpret the variable hindlimb morphology that is preserved in the hominin fossil record. The morphology of the knee in particular has played a central role in palaeoanthropological studies about the form of bipedality our ancestors adopted (Stern & Susman, 1983; Susman, Stern & Jungers, 1984; Crompton et al., 1998; Carey & Crompton, 2005; Lovejoy & McCollum, 2010; Raichlen et al., 2010). Some researchers propose that early hominins, such as australopiths, used bent-hip, bent-knee locomotion, similar to African ape bipedal locomotion (Stern & Susman, 1983; Susman, Stern & Jungers, 1984), while others propose extended-hip and knee locomotion, similar to that of modern humans (Carey & Crompton, 2005; Lovejoy & McCollum, 2010; Raichlen et al., 2010). Studying the morphology of the knee joint and its links to locomotion in extant apes can help reconstruct how early hominins (e.g. australopiths, early *Homo*) walked bipedally, as well as other potential locomotor behaviours in which they may have engaged (e.g. arboreal climbing). However, inferences about the predominant joint posture and locomotion based solely on external morphology are limited by potential phylogenetic lag, in which some features are present but not necessarily functionally significant (Ward, 2002). Recent studies on trabecular bone have demonstrated that this tissue may be more informative for reconstructing joint posture and locomotion during life (Ryan & Ketcham, 2002; Ryan & Shaw, 2012; Tsegai et al., 2013, 2018; Skinner et al., 2015) and provides additional evidence that can improve our understanding of locomotor behaviour in extinct taxa. In this study, we investigate correlations between trabecular bone patterning and knee joint position during locomotion in humans and great apes.

Trabecular bone is a porous structure composed of struts, located in the epiphyses of long bones, as well as short bones, such as carpals and tarsals (Keaveny et al., 2001). It functions physiologically as a mineral reserve, contributing to maintenance of homeostasis through resorption and deposition of bone (Rodan, 1998; Clarke, 2008). Although the mechanical function of trabecular bone is not fully understood, previous studies have demonstrated that its structure transfers joint load from subchondral bone toward the diaphyseal cortical bone (Currey, 2002; Barak, Weiner & Shahar, 2008). Through a process known as bone functional adaptation (Ruff, Holt & Trinkaus, 2006), trabecular structure has been shown to model in relation to the direction and magnitude of load, resulting in changes in overall bone volume as well as the orientation of the trabecular struts (Biewener et al., 1996; Rodan, 1997; Mittra, Rubin & Qin, 2005; Pontzer et al., 2006; Barak, Lieberman & Hublin, 2011; Harrison et al., 2011). Bone volume fraction (ratio of bone volume to total volume, or BV/TV) and degree of anisotropy (DA) can together explain up to 97% of trabecular bone strength (Goulet et al., 1994; Maquer et al., 2015). Other trabecular parameters, such as trabecular number, trabecular separation and trabecular thickness help to describe potential variation in the architecture related to trabecular bone function. Trabecular number, separation and thickness are also linked to overall trabecular bone mechanical strength (Kleerekoper et al., 1985; McCalden, McGeough & Court-Brown, 1997) and to bone quality, as their decline is main contributor to age-related trabecular bone loss (Parfitt et al., 1983; Weinstein & Hutson, 1987). Furthermore, these parameters, in contrast to BV/TV and DA, have been shown to scale allometrically with body size (Doube et al., 2011; Ryan & Shaw, 2013; Barak, Lieberman & Hublin, 2013) and to differ in smaller compared to larger mammals (Barak, Lieberman & Hublin, 2013).

Previous research has revealed a correlation between trabecular patterns and variation in locomotor loading in the proximal femur (Ryan & Ketcham, 2002; Scherf, 2008; Ryan & Shaw, 2012; Ryan et al., in press), the hip and proximal tibia (Volpato et al., 2008; Mazurier, Nakatsukasa & Macchiarelli, 2010) and the ankle of primates (Barak, Lieberman & Hublin, 2013; Tsegai et al., 2017). Longitudinal studies of trabecular bone ontogeny in humans have shown an association with bone modelling and the gait changes that occur with the development of bipedalism (Ryan & Krovitz, 2006; Gosman & Ketcham, 2009; Raichlen et al., 2015; Milovanovic et al., 2017). Looking at the knee specifically, alterations in the orientation of joint position and resulting load were found to correlate with trabecular strut alignment in guinea fowls (Pontzer et al., 2006). Furthermore, compared to a control group, the dominant knees of Olympic fencing athletes were found to have greater BV/TV and trabecular number, but lower trabecular separation, consistent with higher loading (Chang et al., 2008). Saers et al. (2016) found a correlation between mobility levels and trabecular architecture throughout the human lower limb, including the knee, across three human populations. A more recent study found sex differences in subchondral trabecular bone spacing in the knee of humans, with males having more evenly-spaced trabeculae compared to females (Sylvester & Terhune, 2017).

Despite the support for trabecular bone functional adaptation, some studies that focused on a single region of the proximal femur (Ryan & Walker, 2010; Shaw & Ryan, 2012) and the distal femoral metaphysis (Carlson, Lublinsky & Judex, 2008; Wallace et al., 2013) did not detect a clear locomotor signal. These results suggest that non-mechanical factors may affect or constrain trabecular structure and that DA may not necessarily be indicative of variability in locomotor mode. There are multiple other factors that can affect trabecular structure, such as genetic or systemic differences (Paternoster et al., 2013; Tsegai et al., 2018), age and hormone levels (Simkin, Ayalon & Leichter, 1987; Suuriniemi et al., 2004), all of which can obscure functional signals. Furthermore, it is not well understood what prompts modelling and how trabecular bone reacts when loaded (Wallace et al., 2014). However, analysing a single sub-volume may lead to non-homologous bone being sampled across species and may not capture the full structural complexity of the epiphysis (Fajardo & Müller, 2001; Kivell et al., 2011; Lazenby et al., 2011). Several studies have demonstrated that subchondral distribution of trabecular bone can provide important insights into bone loading that are overlooked with a centrally-placed volume of interest; particularly in morphologically complex bones and joints (Tsegai et al., 2013; Skinner et al., 2015; Stephens et al., 2016; Sylvester & Terhune, 2017; Tsegai et al., 2018). In this study, we aim to investigate the trabecular structure throughout the entire distal femoral epiphysis of humans and great apes and how potential variation in this structure might reflect differences in knee joint loading during a variety of locomotor behaviours.

### Locomotion, morphology and predicted knee posture/loading

**Figure 1:**
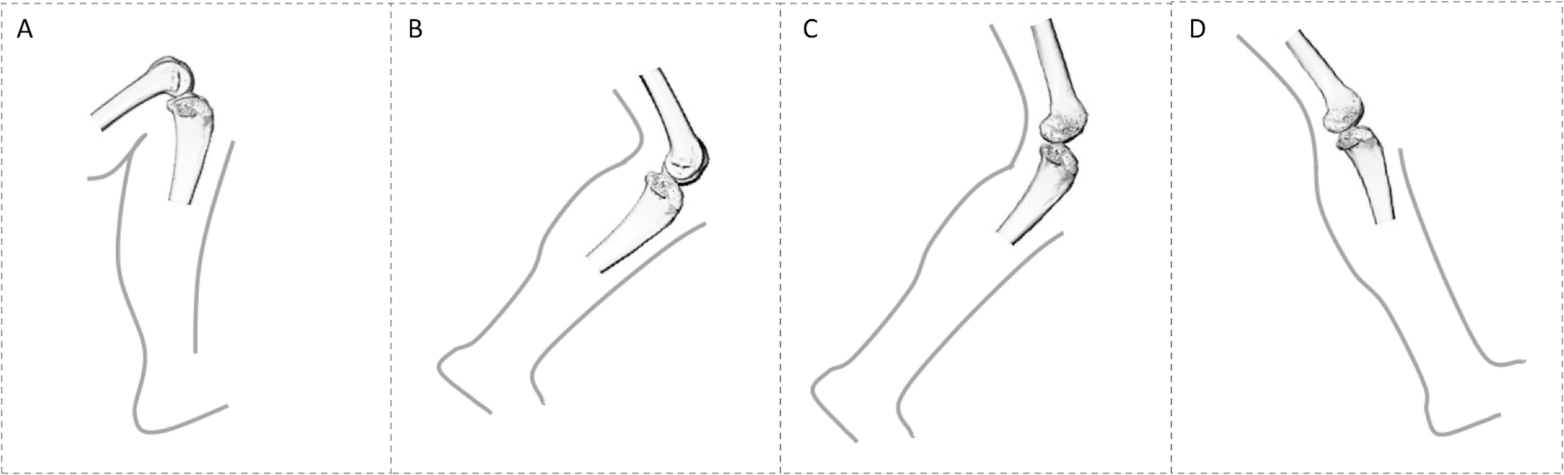
Comparison of knee posture during different habitual locomotor activities in great apes (A–B) and humans (C–D). (A) Great ape knee posture in maximum knee flexion (∼50°) during climbing (Isler, 2005). (B) Great ape knee posture at toe-off (∼120°) during terrestrial knuckle-walking (Finestone et al., 2018). (C) Human knee posture at toe-off (∼145°). (D) Human knee posture at heel-strike (∼160°). These were selected depending on when GRF is highest. In this study, all great apes are considered to show similar degrees of knee flexion during quadrupedal walking, as demonstrated by Finestone et al. (2018) and during climbing, but it should be noted that *Gorilla* has been shown to use a less flexed knee posture during vertical climbing compared with *Pan* (Isler, 2005).

The most frequent locomotor behaviour in *Pan* is quadrupedal knuckle-walking, but they also engage in several other terrestrial as well as arboreal behaviours, including vertical climbing, leaping, bipedalism and suspension (Hunt, 1992; Bauer, 1977; Doran, 1993, 1997; Isler, 2005), where the knee is flexed to varying degrees (D’Août et al., 2004; Isler, 2005; Ankel-Simons, 2007; Pontzer, Raichlen & Sockol, 2009; Lee et al., 2012). During terrestrial knuckle-walking the knee joint angle ranges from ∼161.4° at foot touchdown to ∼117.4° at toe-off (Finestone et al., 2018), and there is inter-individual variation in vertical ground reaction force (GRF). Some individuals show a single vertical GRF peak across the stance phase and others show two distinct peaks, one during early stance and one during late stance (Pontzer, Raichlen & Rodman, 2014). During climbing and jumping they may utilise their full flexion–extension range at the knee (D’Août et al., 2002; Isler, 2005) (Fig. 1).

*Gorilla* also engages most frequently in terrestrial knuckle-walking and practices variable degrees of arboreality, depending on their habitat and body size (Tutin & Fernandez, 1985; Kuroda, 1992; Remis, 1994; Doran, 1996, 1997; Isler, 2005; Crompton, Sellers & Thorpe, 2010; Tocheri et al., 2011). During terrestrial knuckle-walking, knee angles vary from 163.2° at foot touchdown to ∼126.6° at toe-off (Finestone et al., 2018) and adult females, as well as subadults of both sexes, climb with higher frequency than larger males (Isler, 2002, 2005). Additionally, flexion–extension range at the hip has been shown to differ more than 30° between sexes (Hammond, 2014), which would affect knee joint angle as well. Furthermore, range of motion at the knee joint differs between *Gorilla* and *Pan* during terrestrial locomotion and climbing with *Gorilla* practising slightly more extended knee postures (Hofstetter & Niemitz, 1998; Isler, 2005; Crompton, Vereecke & Thorpe, 2008; but see Finestone et al., 2018).

*Pongo* is the most arboreal of the great apes. They are distinguished from African apes by their greater use of torso-pronograde (i.e. quadrumanus suspension) and orthograde suspensory locomotion, and they employ a diversity of positional behaviours when navigating complex arboreal canopies (Thorpe & Crompton, 2005, 2006; Thorpe, Holder & Crompton, 2009). The frequent use of arboreal behaviours, where multiple limbs are used variously to achieve balance (Thorpe & Crompton, 2006; Payne et al., 2006; Thorpe, Holder & Crompton, 2009), alters the distribution of load across the upper and lower limb joints. *Pongo* has also been observed using bipedality and hindlimb suspension, which involves either suspension from both legs with joints extended, suspension from one leg, or suspension from one leg with support from a forelimb (Thorpe & Crompton, 2005, 2006). While climbing is observed in all nonhuman apes and the imposed stresses are similar to bipedal walking (Fleagle et al., 1981), the kinematics of isolated joints differ across species, with *Pongo* showing significantly larger ranges of motion in the hindlimb joints than both gorillas and bonobos (Morbeck & Zihlman, 1988; Tuttle & Cortright, 1988; Isler, 2005). However, the flexion–extension range at the knee during quadrupedal locomotion may not differ significantly to that of African apes (Finestone et al., 2018; mean values are 149.3° at touchdown and 113° at toe-off).

Humans are the only obligate bipedal ape and are unique in that both hips and knees remain relatively extended during the gait cycle (Alexander, 1991, 2004). During the stance phase in human walking, following initial foot contact with the ground, body weight is rapidly transferred to the contacting limb and GRF reaches a maximum (Racic, Pavic & Brownjohn, 2009). The joint angle of the knee during foot touchdown ranges from 170° to 160° (Lafortune et al., 1992; Wallace et al., 2018) (Fig. 1). During midstance the vertical GRF decreases, but the supporting leg carries all of the weight of the individual. While the opposite leg swings and weight is transferred forward, the heel of the supporting limb starts to rise and leads to a second peak of vertical GRF at toe-off (Racic, Pavic & Brownjohn, 2009). The joint angle of the knee at toe-off is approximately 140° (Lafortune et al., 1992). Humans engage in many other bipedal activities, such as running, jumping or squatting, in which and knee flexion/extension can vary considerably. Flexion angles increase during running, reaching 145° at touchdown, while the degree of flexion is greater and differs significantly to walking (Mann & Hagy, 1980). Compared with walking, there is only one (rather than two) peak of vertical GRF during a shorter stance phase and the vertical GRF are substantially higher during running (Nilsson & Thorstensson, 1989; Racic, Pavic & Brownjohn, 2009). Given that we do not know about the types of activities in which our human sample engaged during life, we make the assumption in this study that loading of the distal femur occurs primarily through walking, although recognise that these higher-impact activities, especially if occurring frequently, may also be reflected in the trabecular structure of distal femur.

In addition to differences in joint kinematics and frequency of specific types of locomotion, variation in hominoid knee joint morphology may influence the distribution of load across the condyles of the distal femur and subsequently the trabecular structure. In humans the knee joint is larger relative to body size (Jungers, 1988) and the overall shape of the epiphysis is more square compared with the smaller and more mediolaterally-expanded epiphysis in other hominoids (Tardieu, 1981). Furthermore, the condyles in humans are more equally-sized and the lateral condyle is elliptical, which increases the radius of curvature and favours extension of the knee (Heiple & Lovejoy, 1971; Tardieu, 1981). In contrast, in *Gorilla*, *Pan* and *Pongo*, the articular surface of the medial condyle is larger than that of the lateral and the condyles are more circular. The disparity in relative condylar size results in increased mediolateral rotation in nonhuman apes at different stages of gait, whereas in humans mediolateral rotation is restricted to the final stage of the flexion–extension cycle, which “locks” the knee during extension (Tardieu, 1981). The varus angle of the ape femur results in higher loading of the medial condyle, while the valgus angle in humans transfers the line of load relatively closer to the lateral condyle, resulting in more equal loading of the two condyles during stance (Preuschoft & Tardieu, 1996).

### Hypotheses

This study will investigate potential variation in the trabecular structure of the human and great ape distal femur, focusing primarily on BV/TV and DA, as well as architectural differences in trabecular number (Tb.N), trabecular separation (Tb.Sp) and trabecular thickness (Tb.Th), and how this variation relates to different locomotor and morphological traits across hominoids. Specifically, we test the following hypotheses:

1. BV/TV distribution will reflect knee joint positioning during habitual locomotion (Fig. 1) and will differ across genera. Specifically, although *Homo* is predicted to have comparatively lower BV/TV values overall (Chirchir et al., 2015, 2017; Ryan & Shaw, 2015), BV/TV distribution will be concentrated distally beneath the condylar articular surfaces, spanning from the medial and lateral grooves to the posteroinferior region of the condyles, to reflect the habitual use of a more extended knee posture during bipedalism. Thus, we expect that high BV/TV will be detected in the distal and posteroinferior regions of the condyles. *Pan* and *Gorilla* are predicted to exhibit greater BV/TV in the posteroinferior and posterosuperior regions of the condyles to reflect more flexed knee postures during quadrupedal knuckle-walking and, particularly, climbing. Vertical climbing mechanics have been studied in bonobos (Isler, 2005), but have not yet been quantified in chimpanzees, thus for the purpose of this study both *Pan* species are assumed to be similar. *Pongo* is predicted to have a more homogenous distribution of BV/TV throughout the condyles and high BV/TV extending from the distal to the posterosuperior region of the condyles, reflecting more variable knee joint postures and loading during their more complex locomotor repertoire.
2. DA distribution will reflect differences in habitual range of motion and loading of the knee joint in particular postures. *Homo* will display the highest DA in the distal and posteroinferior regions of the condyles, resulting from the stereotypical loading of these regions during bipedal locomotion and their overall less mobile knee joints relative to other apes (Tardieu, 1981). *Pan* and *Gorilla* will exhibit similar DA patterns, with lower values than *Homo* specifically in the posterior regions of the condyles, due to increased rotational movement of their knees during locomotion (Tardieu, 1981) and higher loading of the posterior when utilising flexed knee postures. *Pongo* will display the lowest DA within the medial and lateral condyles in all studied regions, due to their more mobile knee joints and varied locomotor loading regime, which results in varied loading of the different regions of the condyles.
3. Architectural variables Tb.N, Tb.Sp and Tb.Th will reflect variation in body size, as demonstrated in previous studies (Doube et al., 2011; Ryan & Shaw, 2013; Barak, Lieberman & Hublin, 2013), and be consistent with potential variation in BV/TV across taxa. Specifically, Tb.N is expected to be higher in smaller-bodied *Pan* and *Pongo* and lower in larger-bodied *Homo* and *Gorilla* across studied regions, while Tb.Sp and Tb.Th are expected to present the opposite pattern. Allometric relationships were not directly analysed due to small and unbalanced sample sizes of each taxon, however they are assumed to follow the same patterns found in previous studies of the femur, and other long bones, across larger samples of primates (Ryan & Shaw, 2013; Barak, Lieberman & Hublin, 2013; Tsegai et al., 2013; Fajardo et al., 2013) and mammals (Doube et al., 2011; Barak, Lieberman & Hublin, 2013).

## Materials and Methods

### Sample and scanning

The study sample is summarised in Table 1. The *Pan troglodytes verus* sample (*n* = 18) is from the Taï Forest collection of the Max Planck Institute for Evolutionary Anthropology in Leipzig, Germany. The *Gorilla gorilla gorilla* sample (*n* = 14) is from the Powell-Cotton Museum, UK of which 13 are from Cameroon and one is from the Democratic Republic of the Congo. The *Pongo* sample (*n* = 7) is from the Zoologische Staatssammlung München, Germany. Five individuals are *Pongo pygmaeus*, one *is P. abelii* and the species of one individual is unknown. The *Homo sapiens* sample (*n* = 11) is from the anthropology collection of Georg-August-Universität Göttingen, Germany and comes from two sub-collections. One of the specimens is from an early 1900s population from a cemetery in Inden that was used between 1877 and 1924 and ten specimens are from a cemetery in Göttingen that was used between 1851 and 1889. There is no additional information on the sample. All nonhuman apes in the study sample were wild shot, except two captive *Pongo* specimens (the only male in the sample and one female). All statistical analyses were repeated excluding the two captive individuals to test for potential bias (see below). All individuals were adult, based on epiphyseal fusion of the femur and associated skeletal elements, and none showed signs of pathologies.

**Table 1:** Taxonomic composition of the study sample, voxel size range (after resampling), sex distribution and microCT scanning parameters.

Taxon	Locomotor mode	*N*	Voxel size (mm)	Sex	Scanning
*Pan troglodytes verus*	Arboreal/knuckle-walker	18	0.040	11 female, five male, two unknown	kV: 120–150, μA: 80–120, 0.25 or 0.5 mm brass
*Gorilla gorilla gorilla*	Terrestrial knuckle-walker	14	0.048–0.089	Seven female, seven male	kV: 130–180, μA: 100–160, 0.1–0.5 mm copper
*Pongo sp.*	Arboreal/torso-pronograde suspension	7	0.035–0.045	Six female, one male	kV: 140, μA: 140, 0.5 mm brass
*Homo sapiens*	Bipedal	11	0.050–0.065	Three female, seven male, one unknown	kV: 140, μA: 140, 0.5 mm brass

**Figure 2:**
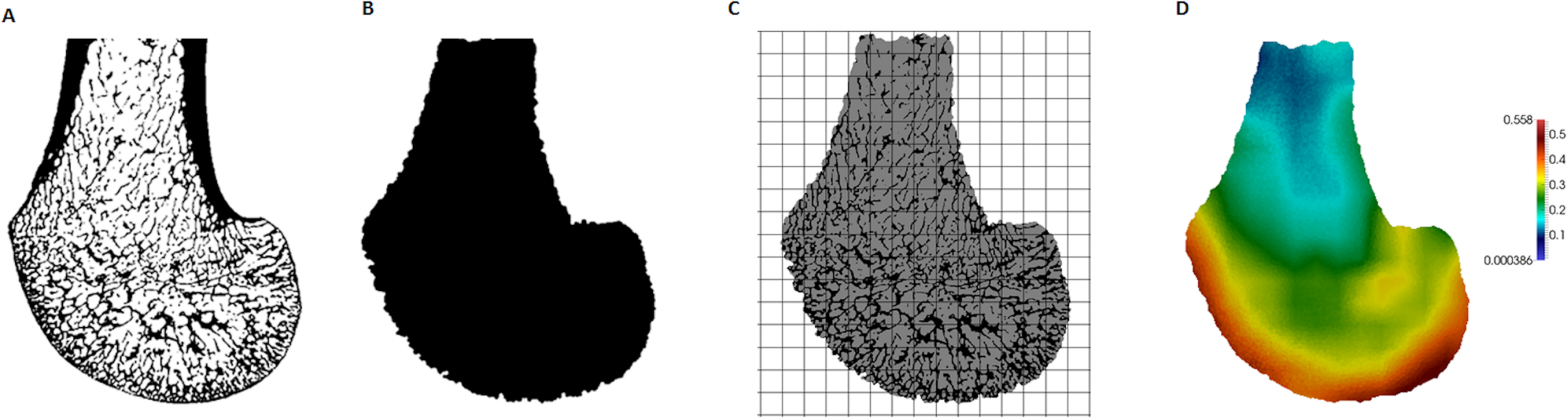
Processing steps of a *Gorilla* specimen, showing a parasagittal view through the lateral condyle. (A) Segmented microCT scan. (B) Inner trabecular area. (C) Trinary mask representing inner air, outer air and trabecular structure, as well as the 3D background grid. (D) BV/ TV distribution within this slice (scaled to its own data range).

*Pan*, *Pongo* and *Homo* samples were scanned using a BIR ACTIS 225/300 industrial microCT scanner housed in the Department of Human Evolution, Max Planck Institute for Evolutionary Anthropology. *Gorilla* specimens were scanned using a Nikon XT 225 ST microCT scanner housed in Cambridge Biotomography Centre, Department of Zoology, at the University of Cambridge. Scans were reconstructed from 1,080 projections into 16-bit TIFF image stacks with isotropic voxel sizes. All scans were oriented to approximate anatomical position in AVIZO 6.3® (Visualization Sciences Group, SAS) to assist comparison. Subsequently, they were cropped and larger scans were re-sampled prior to segmentation to overcome computational limitations. The final range of resolution for each species is detailed in Table 1. The Ray Casting Algorithm (Scherf & Tilgner, 2009) was used to segment bone in all specimens (Fig. 2A).

### Trabecular architecture analysis

A whole-epiphysis approach was used to analyse the patterns of trabecular bone distribution in medtool v4.1 (http://www.dr-pahr.at) following published protocols (Gross et al., 2014). Morphological filters were applied to define and separate cortical from trabecular bone. In regions with marked depressions (or that are c-shaped), separation of the cortical shell from trabecular bone can be less reliable (see Pahr & Zysset, 2009 for explanation). In our study this was specifically an issue within the intercondyloid fossa. In specimens that presented this problem, a correction filter was applied within a manually selected bounding box. This filter re-defines cortical and trabecular bone in the selected volume by applying the algorithm iteratively. The accuracy of the separation was evaluated using AVIZO 6.3® (Visualization Sciences Group, SAS). Nonetheless, the regions of interest, and specifically the condyles, were not affected by this issue. Following the definition of the different anatomical structures, the cortical bone was removed (Fig. 2B). Trabecular thickness values were obtained for each specimen from the isolated trabecular structure using the BoneJ plug-in (version 1.4.1, Doube et al., 2010) for ImageJ (Schneider, Rasband & Eliceiri, 2012) and were used to validate the size of the sphere used in the morphological filters (see Gross et al., 2014).

A mask representing the inner air, outer air and trabecular structure (each with different grey values) was then produced. Both the mask representing the inner region (Fig. 2B) and this trinary mask (Fig. 2C) were used in the following meshing process. A 3D rectangular background grid with a grid size of 3.5 mm was built around each segmented volume (Fig. 2C) and a sampling sphere of 7.5 mm in diameter was used to measure BV/TV and DA at each node using medtool v4.1. DA was calculated as DA = 1 − [smallest eigenvalue/largest eigenvalue], obtained using the mean-intercept-length method (Whitehouse, 1974; Odgaard, 1997). Three-dimensional tetrahedral meshes of all specimens were created with CGAL 4.4 (CGAL, Computational Geometry, http://www.cgal.org), using the segmented trabecular structure and a mesh size of 0.6 mm. The values at each node were then interpolated to the tetrahedral elements and the resulting BV/TV (Fig. 2D) and DA distribution maps were visualised using Paraview v4.0.1 (Ahrens, Geveci & Law, 2005).

**Figure 3:**
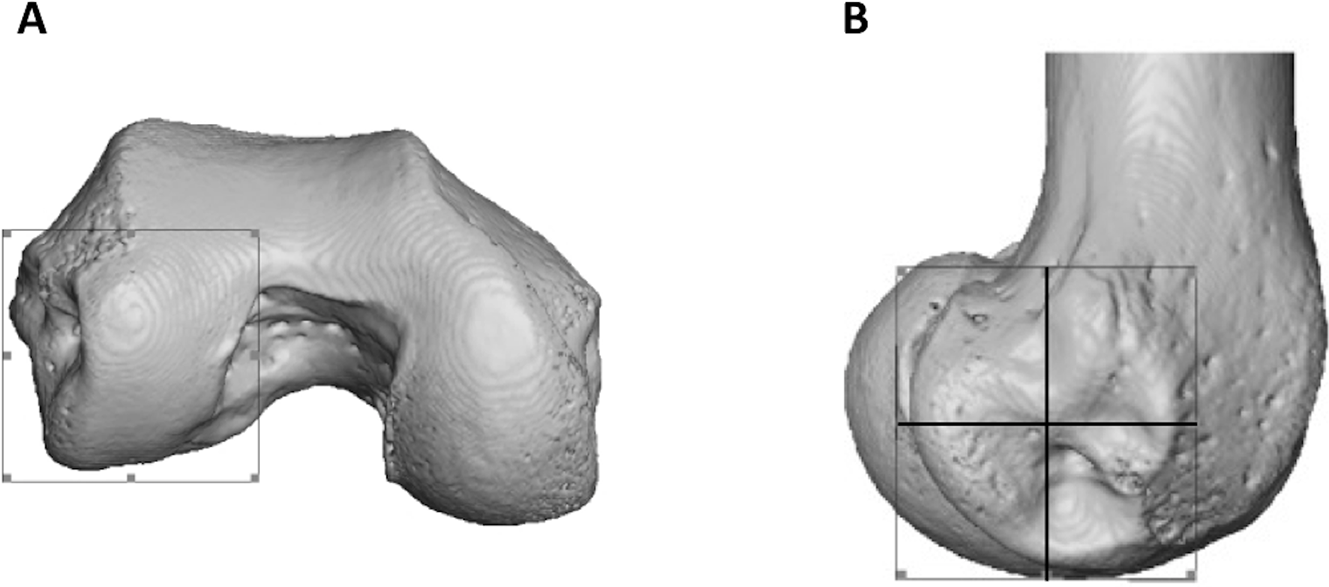
Partitioning of the lateral condyle into sub-regions in a *Pan* specimen. (A) Selection of condyle. (B) Separation into quarters, including the distal (bottom, right), posteroinferior (bottom, left) and posterosuperior (top, left). The anterosuperior quadrant (top, right) was not analysed. The medial condyle was partitioned in the same way.

To statistically test for regional differences in trabecular structure, three subregions of each condyle were isolated (distal, posteroinferior and posterosuperior) in a subsample of 10 individuals from each species (all seven *Pongo* were included). Condyles were defined based on the extent of the articular surface and the patello-femoral articulation was excluded (Fig. 3A). Each condyle was divided into equal quarters using an automated script in medtool v4.1 (Fig. 3B). The anterosuperior quarter of both condyles was excluded from the analysis, as it was not adjacent to the articular surface. Analyses of BV/TV and DA for the sub-regions were repeated as above and Tb.Th and Tb.Sp were calculated for these regions with an in-house script using the Hildebrand & Rüegsegger (1997) method, similar to what is used in BoneJ. Tb.N was calculated as Tb.N = 1/(Tb.Th + Tb.Sp).

### Statistical analysis

All statistical analyses were done in R v3.4.1 (R Core Team, 2017). The Kruskal–Wallis test was used to examine regional differences in all parameters (BV/TV, DA, Tb.N, Tb.Sp, Tb.Th) among taxa, with Wilcoxon rank sum test post-hoc analysis for pairwise comparisons. To further compare regional differences in BV/TV and DA, we calculated an “inferior ratio” comparing the distal and posteroinferior regions, as well as a “posterior ratio” comparing the posteroinferior and posterosuperior regions. These ratios were selected to examine species-specific patterns in BV/TV and DA distribution that may not be revealed when the isolated regions are directly compared between species. Furthermore, all tests were repeated excluding the captive *Pongo* specimens to test for impact of these specimens on the results. A principal components (PC) analysis was conducted to detect which trabecular parameters contribute most to inter-specific differences. DA, Tb.Sp and Tb.Th of all tested regions were included in the PC analysis. We excluded BV/TV and Tb.N from the PC analysis because multivariate regression revealed that both variables were significantly correlated with Tb.Sp and Tb.Th. This was not surprising as Tb.N was calculated using the Tb.Th and Tb.Sp values obtained directly from the specimens and BV/TV is defined by all these parameters.

## Results

### Quantitative and qualitative analysis of trabecular parameters

**Figure 4:**
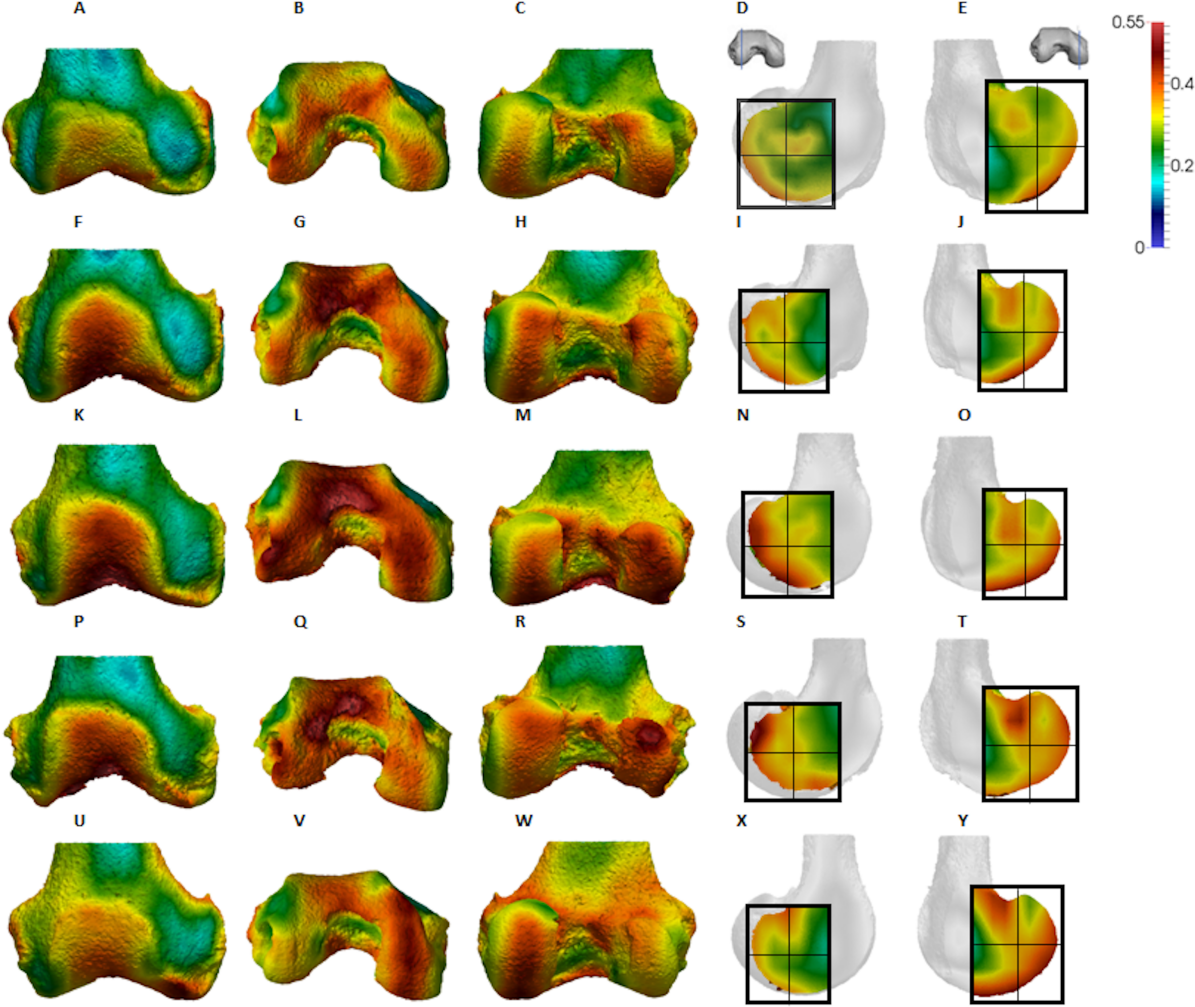
*Pan* BV/TV distribution. (A) Anterior view. (B) Inferior view. (C) Posterior view. (D) Lateral condyle. (E) Medial condyle. (F–J) Specimen MPITC 15001. (F) Anterior view. (G) Inferior view. (H) Posterior view. (I) Lateral condyle. (J) Medial condyle. (K-O) Specimen MPITC 11786. (K) Anterior view. (L) Inferior view. (M) Posterior view. (N) Lateral condyle. (O) Medial condyle. (P–T) Specimen MPITC 11793. (P) Anterior view. (Q) Inferior view. (R) Posterior view. (S) Lateral condyle. (T) Medial condyle. (U–Y) Specimen MPITC 11778. (U) Anterior view. (V) Inferior view. (W) Posterior view. (X) Lateral condyle. (Y) Medial condyle. All specimens are from the right side. In anterior and inferior views the medial condyle is on the right. In the posterior view the medial condyle is on the left. The location of the parasagittal slice through each condyle is indicated above and the main areas of interest are outlined. Individuals are scaled to the same data range.

**Figure 5:**
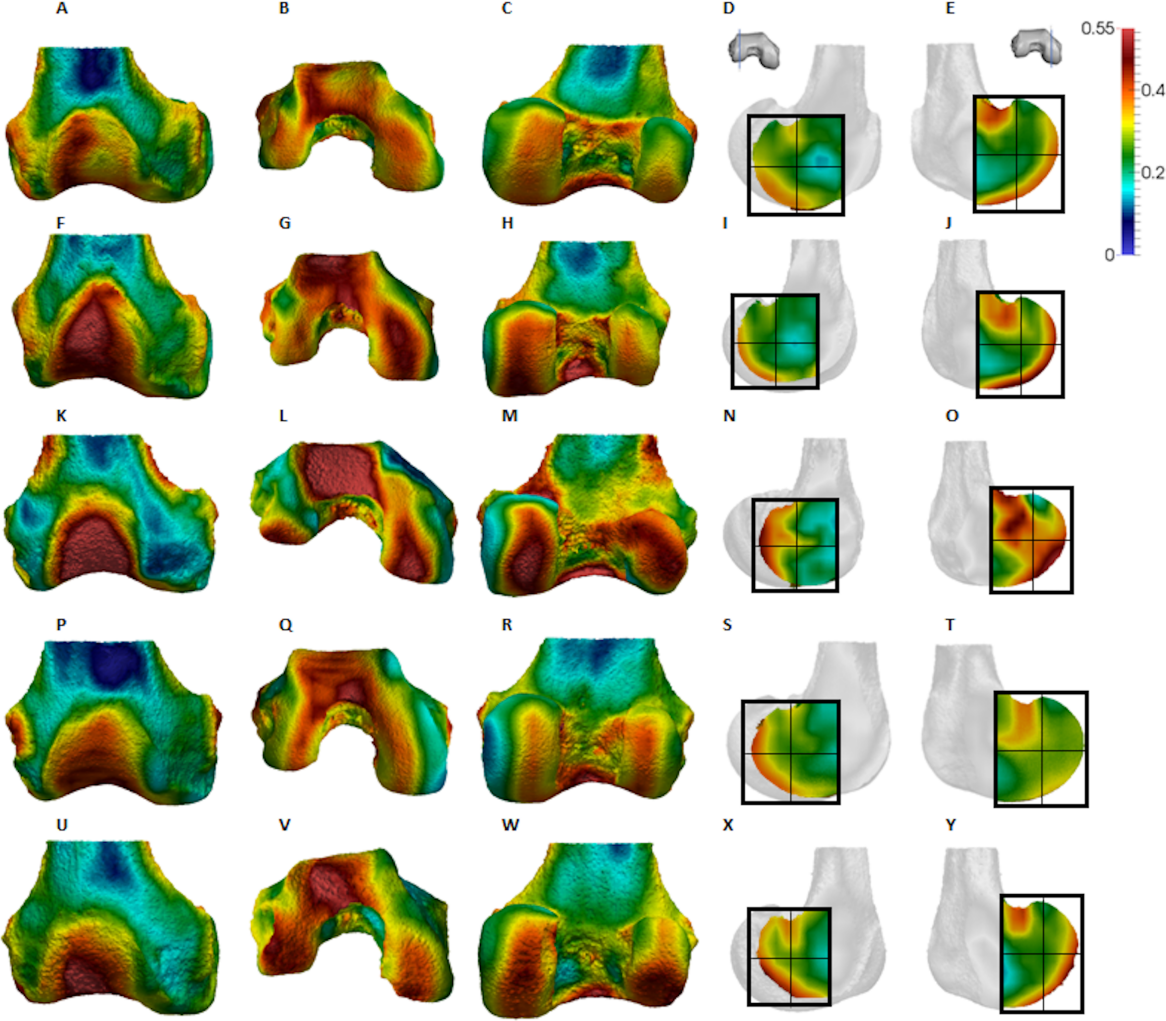
*Gorilla* BV/TV distribution. (A–E) Specimen M95. (A) Anterior view. (B) Inferior view. (C) Posterior view. (D) Lateral condyle. (E) Medial condyle. (F–J) Specimen M300. (F) Anterior view. (G) Inferior view. (H) Posterior view. (I) Lateral condyle. (J) Medial condyle. (K–O) Specimen M372. (K) Anterior view. (L) Inferior view. (M) Posterior view. (N) Lateral condyle. (O) Medial condyle. (P-T) Specimen M798. (P) Anterior view. (Q) Inferior view. (R) Posterior view. (S) Lateral condyle. (T) Medial condyle. (U–Y) Specimen M856. (U) Anterior view. (V) Inferior view. (W) Posterior view. (X) Lateral condyle. (Y) Medial condyle. All specimens are from the right side. In anterior and inferior views the medial condyle is on the right. In the posterior view the medial condyle is on the left. The location of the parasagittal slice through each condyle is indicated above and the main areas of interest are outlined. Individuals are scaled to the same data range.

**Figure 6:**
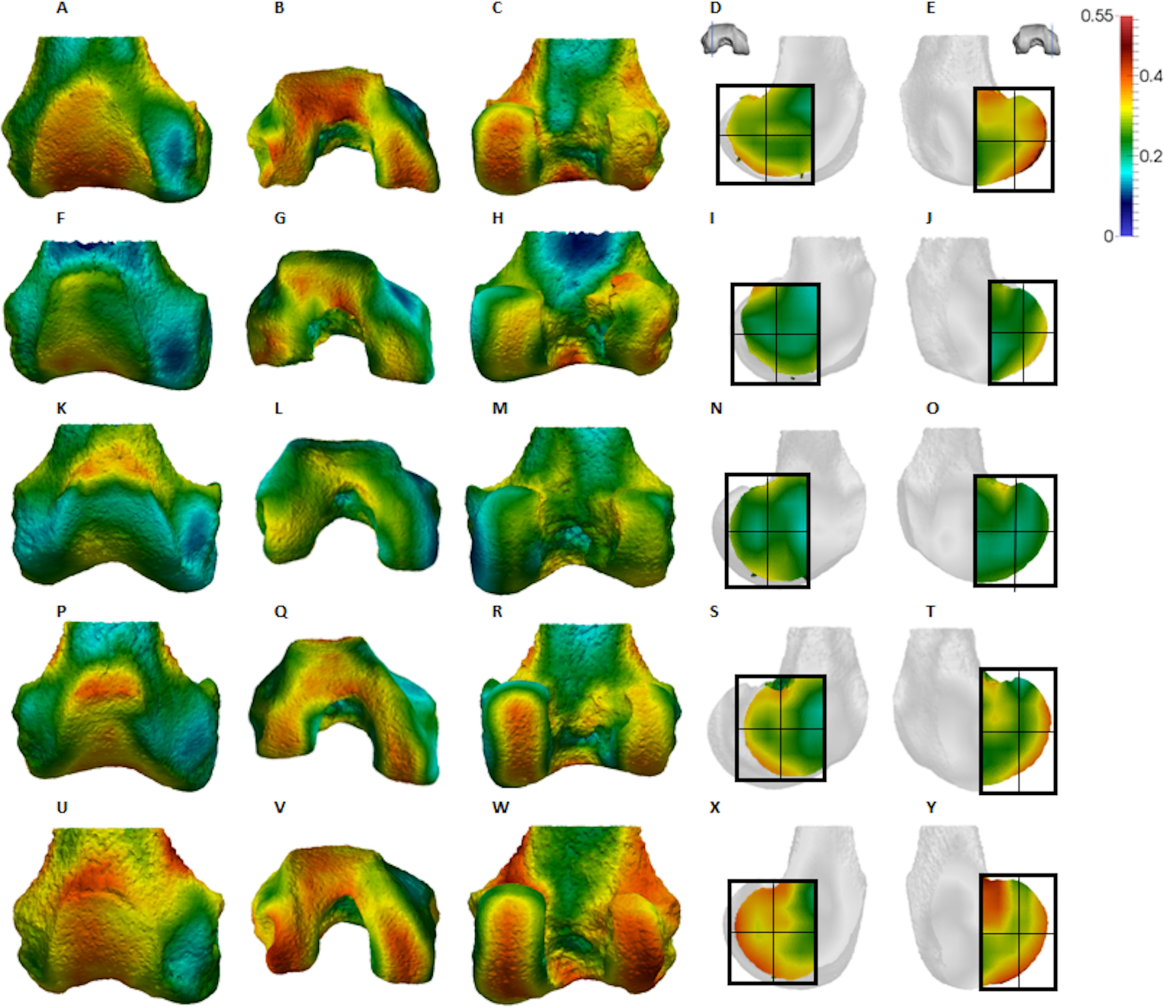
*Pongo* BV/TV distribution. (A–E) Specimen ZSM 1909 0801. (A) Anterior view. (B) Inferior view. (C) Posterior view. (D) Lateral condyle. (E) Medial condyle. (F–J) Specimen ZSM 1907 0660. (F) Anterior view. (G) Inferior view. (H) Posterior view. (I) Lateral condyle. (J) Medial condyle. (K–O) Specimen ZSM 1973 0270. (K) Anterior view. (L) Inferior view. (M) Posterior view. (N) Lateral condyle. (O) Medial condyle. (P–T) Specimen ZSM 1907 0483. (P) Anterior view. (Q) Inferior view. (R) Posterior view. (S) Lateral condyle. (T) Medial condyle. (U–Y) Specimen ZSM 1907 0633B. (U) Anterior view. (V) Inferior view. (W) Posterior view. (X) Lateral condyle. (Y) Medial condyle. All specimens are from the right side. In anterior and inferior views the medial condyle is on the right. In the posterior view the medial condyle is on the left. The location of the parasagittal slice through each condyle is indicated above and the main areas of interest are outlined. Individuals are scaled to the same data range. Captive specimens are not included in the figure but can be found in the Supplemental Files.

**Figure 7:**
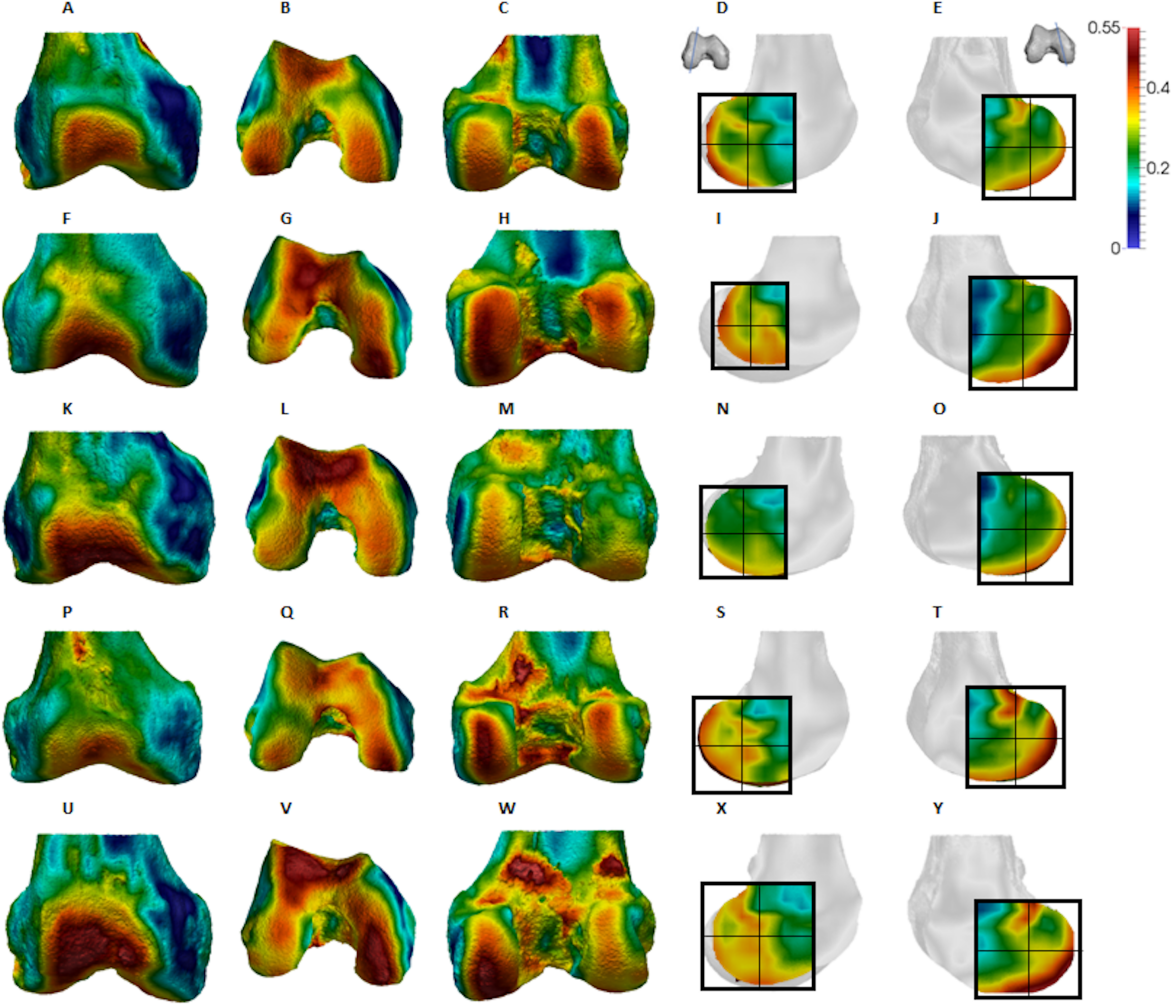
*Homo* BV/TV distribution. (A–E) Specimen Campus 66. (A) Anterior view. (B) Inferior view. (C) Posterior view. (D) Lateral condyle. (E) Medial condyle. (F–J) Specimen Campus 36. (F) Anterior view. (G) Inferior view. (H) Posterior view. (I) Lateral condyle. (J) Medial condyle. (K–O) Specimen Campus 72. (K) Anterior view. (L) Inferior view. (M) Posterior view. (N) Lateral condyle. (O) Medial condyle. (P–T) Specimen Campus 86. (P) Anterior view. (Q) Inferior view. (R) Posterior view. (S) Lateral condyle. (T) Medial condyle. (U–Y) Specimen Campus 81. (U) Anterior view. (V) Inferior view. (W) Posterior view. (X) Lateral condyle. (Y) Medial condyle. All specimens are from the right side. In anterior and inferior views the medial condyle is on the right. In the posterior view the medial condyle is on the left. The location of the parasagittal slice through each condyle is indicated above and the main areas of interest are outlined. In *Homo* the slice is angled as it follows the orientation of the condyles and runs through the centre of each condyle. Individuals are scaled to the same data range.

**Figure 8:**
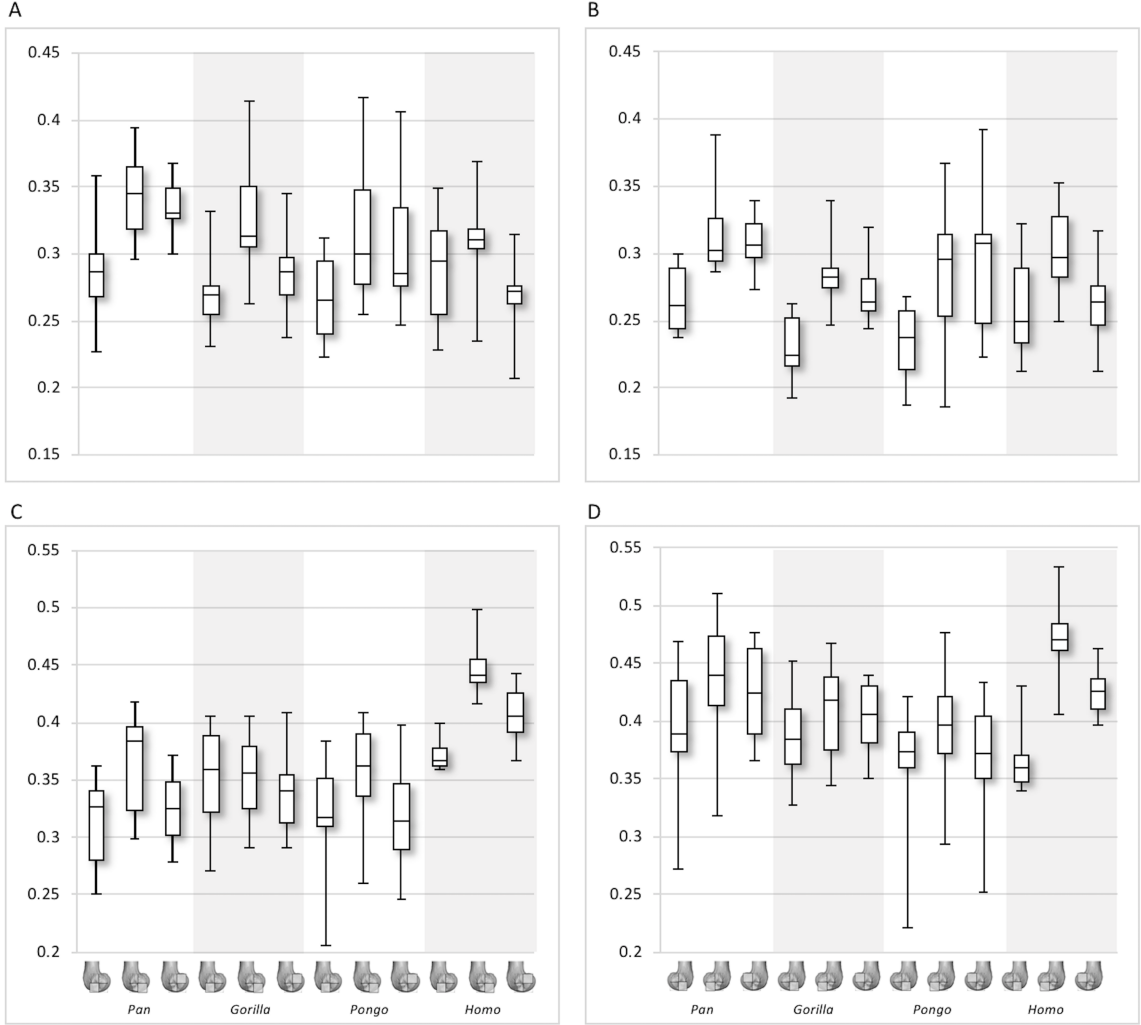
Bone volume fraction (BV/TV) and degree of anisotropy (DA) results for each region and taxon. (A) BV/TV in the lateral condyle. (B) BV/TV in the medial condyle. (C) DA in the lateral condyle. (D) DA in the medial condyle. Regions (outlined) and taxa are displayed below.

**Figure 9:**
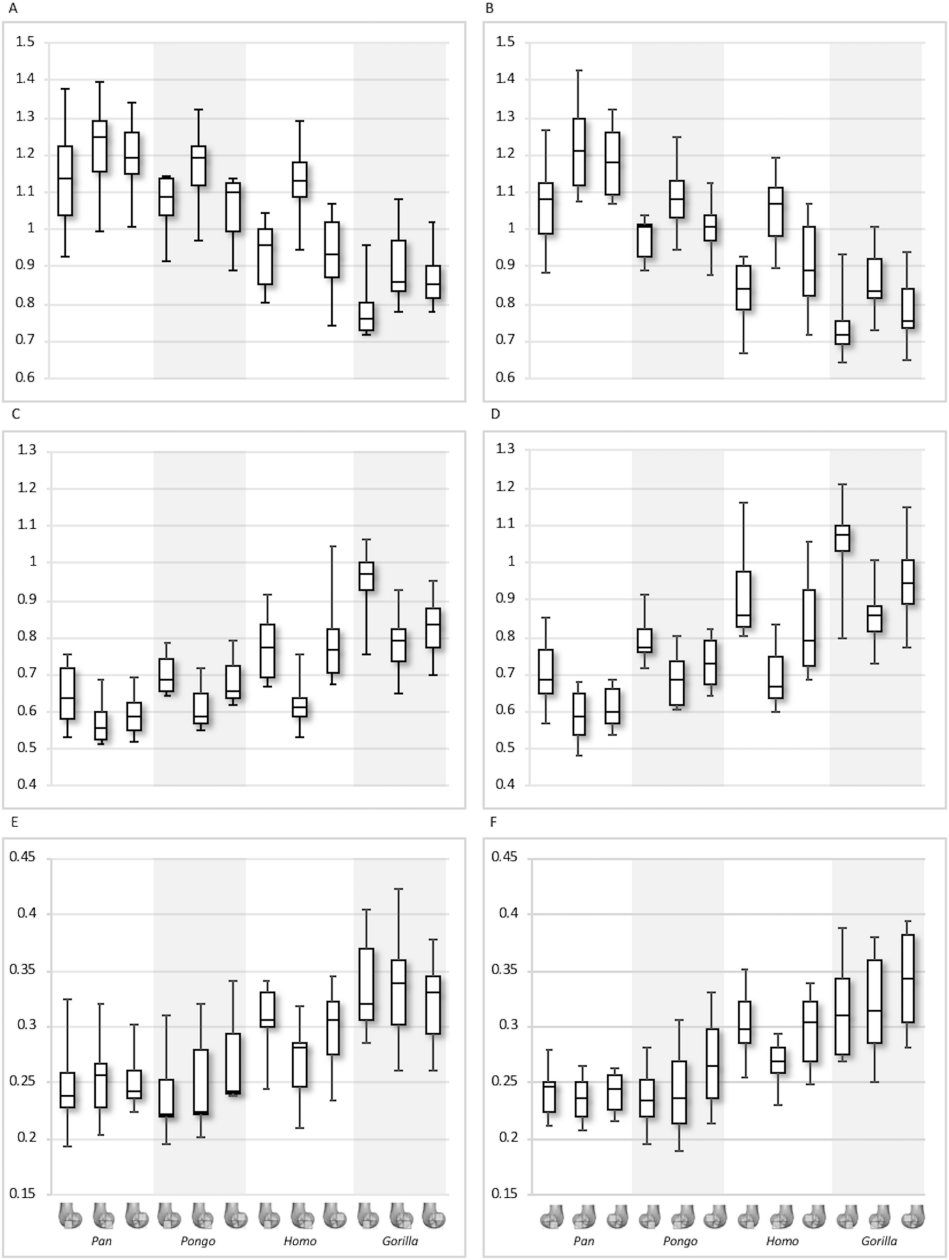
Trabecular number (Tb.N), separation (Tb.Sp) and thickness (Tb.Th) results for each region and taxon. (A) Tb.N in the lateral condyle. (B) Tb.N in the medial condyle. (C) Tb.Sp in the lateral condyle. (D) TB.Sp in the medial condyle. (E) Tb.Th in the lateral condyle. (F) Tb.Th in the medial condyle. Regions (outlined) and taxa are displayed below. Taxa are presented in order of body mass (*Pan* the smallest; *Gorilla* the largest) to better visualise any patterns potentially associated with body size.

Quantitative and qualitative analysis of the trabecular architecture in the distal femur reveal differences across taxa. Figures 4–7 present BV/TV distribution in five individuals of each taxon and the Supplemental Information contains images for each specimen in the study sample. Quantitative results are shown in Figures 8–9 and are detailed in Table 2 and Table S1. Analyses were repeated excluding the two *Pongo* captive specimens and since in most cases the results did not change, the specimens were included in the analysis (when differences were found, they are reported below).

**Table 2:** Trabecular architecture results by condyle and region.

Taxon	Parameter	Lateral distal	CV	Lateral posteroinferior	CV	Lateral posterosuperior	CV	Medial distal	CV	Medial posteroinferior	CV	Medial posterosuperior	CV
*Pan*	BV/TV	0.29 (0.04)	13.1	0.34 (0.03)	9.8	0.33 (0.02)	6.5	0.27 (0.03)	9.3	0.32 (0.03)	10.1	0.31 (0.02)	6.3
	DA	0.31 (0.04)	4.0	0.37 (0.04)	11.7	0.33 (0.03)	9.7	0.39 (0.06)	15.2	0.43 (0.06)	13.7	0.43 (0.04)	9.8
	Tb.N (1/mm)	1.14 (0.14)	12.3	1.22 (0.12)	9.7	1.20 (0.09)	7.8	1.07 (0.12)	11.4	1.22 (0.12)	9.8	1.18 (0.10)	8.2
	Tb.Sp (mm)	0.65 (0.08)	12.7	0.57 (0.06)	10.1	0.59 (0.05)	8.8	0.70 (0.09)	12.9	0.59 (0.07)	11.3	0.61 (0.05)	8.8
	Tb.Th (mm)	0.25 (0.04)	14.4	0.25 (0.03)	13.1	0.25 (0.02)	9.1	0.24 (0.02)	9.2	0.24 (0.02)	8.4	0.24 (0.02)	7.3
*Gorilla*	BV/TV	0.27 (0.03)	10.6	0.33 (0.05)	13.6	0.29 (0.04)	12.4	0.23 (0.02)	9.6	0.29 (0.03)	9.4	0.27 (0.02)	8.5
	DA	0.35 (0.05)	4.5	0.35 (0.04)	10.5	0.34 (0.03)	10.0	0.39 (0.02)	5.8	0.41 (0.04)	10.4	0.40 (0.03)	7.6
	Tb.N (1/mm)	0.78 (0.07)	9.5	0.90 (0.10)	10.6	0.87 (0.08)	8.6	0.74 (0.08)	11.0	0.86 (0.08)	9.4	0.78 (0.08)	10.6
	Tb.Sp (mm)	0.95 (0.08)	8.4	0.78 (0.08)	9.8	0.83 (0.08)	9.5	1.06 (0.11)	10.4	0.86 (0.07)	8.4	0.95 (0.10)	10.8
	Tb.Th (mm)	0.34 (0.04)	12.6	0.34 (0.05)	15.8	0.32 (0.04)	12.0	0.32 (0.04)	13.5	0.32 (0.05)	14.7	0.34 (0.04)	12.3
*Pongo*	BV/TV	0.27 (0.04)	13.2	0.32 (0.06)	17.7	0.31 (0.05)	17.6	0.23 (0.03)	13.6	0.28 (0.06)	20.7	0.29 (0.06)	19.8
	DA	0.32 (0.06)	5.8	0.36 (0.05)	14.5	0.32 (0.05)	15.6	0.36 (0.07)	18.1	0.39 (0.06)	15.0	0.37 (0.06)	16.2
	Tb.N (1/mm)	1.07 (0.09)	8.1	1.17 (0.11)	9.8	1.05 (0.09)	8.9	0.97 (0.06)	6.0	1.09 (0.10)	9.2	1.01 (0.08)	7.6
	Tb.Sp (mm)	0.70 (0.06)	8.5	0.61 (0.07)	10.6	0.69 (0.07)	10.2	0.79 (0.06)	8.1	0.69 (0.08)	11.1	0.73 (0.07)	9.6
	Tb.Th (mm)	0.24 (0.04)	17.1	0.25 (0.05)	4.6	0.27 (0.05)	16.5	0.24 (0.03)	12.9	0.24 (0.05)	18.7	0.27 (0.04)	16.3
*Homo*	BV/TV	0.29 (0.04)	14.2	0.31 (0.04)	11.3	0.27 (0.03)	12.2	0.26 (0.04)	14.8	0.30 (0.03)	11.2	0.26 (0.03)	11.4
	DA	0.37 (0.01)	1.3	0.45 (0.02)	5.1	0.41 (0.03)	6.0	0.37 (0.04)	10.4	0.47 (0.03)	7.1	0.43 (0.02)	4.7
	Tb.N (1/mm)	0.93 (0.09)	9.6	1.12 (0.11)	9.6	0.93 (0.11)	12.3	0.83 (0.08)	9.8	1.05 (0.10)	9.5	0.91 (0.12)	12.8
	Tb.Sp (mm)	0.78 (0.09)	12.1	0.63 (0.07)	10.9	0.80 (0.12)	15.4	0.91 (0.12)	12.8	0.69 (0.09)	12.3	0.82 (0.13)	15.4
	Tb.Th (mm)	0.31 (0.03)	9.8	0.27 (0.03)	12.0	0.30 (0.03)	11.5	0.30 (0.03)	9.9	0.27 (0.02)	8.0	0.30 (0.03)	11.1

**Note:**

Mean values, standard deviation and coefficient of variation are included for each parameter and region.

Qualitative comparison reveals the variability in distribution patterns across taxa, while quantitative comparison reveals differences in BV/TV values in specific regions. *Pan* shows high BV/TV extending deep to the articular surface of the condyles, from the medial and lateral grooves to the posteriorsuperior margin of both condyles (Fig. 4). This is consistent in all the specimens and is most pronounced on the medial condyle. *Gorilla* and *Pongo* present a similar pattern to that of *Pan* with regions of high BV/TV that extend from the inferior margin of the patellar articulation to the posterior region of both condyles (Figs. 5 and 6). However, in *Gorilla* this high concentration does not extend as posterosuperiorly as in *Pan*. Also, in the medial condyle high BV/TV does not extend as anteriorly as it does in *Pan*. In *Gorilla*, the distribution of BV/TV along the lateral condyle is more variable across individuals. *Homo* show a greater range of BV/TV values, indicated by their higher CV (coefficient of variation) (Table 2), and their range overlaps with the other species. Humans generally show high BV/TV in the posteroinferior region of the condyles, which in some individuals extends further posterosuperiorly (Fig. 7). In the lateral condyle they also show high BV/TV in the distal region. Generally, the apes appear to have lower BV/TV in the distal region of both condyles compared to humans (Fig. 8). No differences in BV/TV are found between species in the inferior regions, but significant differences are found in the posterosuperior region in both condyles. *Pan* shows significantly higher BV/TV in this region than both *Gorilla* (lateral *p* < 0.05; medial *p* < 0.01) and *Homo* (lateral *p* < 0.001; medial *p* < 0.05), but the *Pan* range overlaps with that of *Pongo*. In the posterior regions of both condyles, *Pongo* have the highest CV values, indicating that they have the most variable trabecular structure. Qualitative analysis shows that in *Pongo*, there is a consistent distribution of high BV/TV values over the posterosuperior margin of both condyles, where the gastrocnemius heads originate (Diogo et al., 2013a); this concentration is occasionally found in African apes.

The qualitative data (Figs. 4–7) reveal differences deep to the patellar articular surface, that were not tested for significant differences in the quantitative comparison. *Pan* shows high BV/TV concentrations centrally and inferiorly, suggesting loading of this surface during knee flexion. Farther from the articular surfaces and within the shaft, BV/TV values decrease. In *Gorilla* high values are distributed evenly across the surface, but there is not a consistent pattern of distribution across all individuals. In *Pongo* the pattern of distribution is variable, with some specimens showing high BV/TV values over the superior margin of the articulation while in others the highest BV/TV is more central and inferior. Lastly in *Homo*, some individuals show high BV/TV on the lateral patellar articular surface, in agreement with valgus knee loading, however this is not consistent across specimens.

Quantitative results also show significant between-species differences in DA (Fig. 8). In the lateral condyle, *Homo* have significantly higher DA in the distal region than *Pan* (*p* < 0.001), but not the other taxa. In the posterior regions of this condyle, *Homo* differ significantly from all other apes (all *p* < 0.001, except the posteroinferior region with *Gorilla* and *Pongo p* < 0.01), showing consistently higher DA values than the other taxa. In the medial condyle, significant differences are only found in the posteroinferior region. *Homo* shows significantly higher DA in this region than both *Gorilla* (*p* < 0.05) and *Pongo* (*p* < 0.05), but not *Pan*. No significant difference is found between the nonhuman apes. *Pongo* shows the most variability in DA values across regions and consistently have the highest CV values, contrary to *Homo* which are the least variable. However, when the captive specimens are removed, the difference between *Homo* and *Pongo* is no longer significant. Variation in the DA distribution can be seen in central parasagittal slices through the condyles, provided for the whole sample in the Supplementary Online Material (S1–S4).

Interspecific differences are also detected in Tb.N, Tb.Sp and Tb.Th (Fig. 9). In both condyles, Tb.N shows a decreasing trend from *Pan* to *Pongo* to *Homo* and to *Gorilla*, which is consistent with increases in body mass. In the lateral condyle, *Gorilla* has significantly lower Tb.N than all other apes in all regions (*Pan p* < 0.001, *Pongo p* < 0.01 in the inferior regions and *p* < 0.05 in the posterosuperior, *Homo p* < 0.05), except *Homo* in the posterosuperior region. *Homo* do not show significant differences with *Pongo* in any region, but when the captive specimens are removed there is a weak but significant result (*p* = 0.05) in the distal and posterosuperior regions. *Homo* also displays significantly lower Tb.N than *Pan* in the distal (*p* < 0.05) and posterosuperior (*p* < 0.001) regions of the lateral condyle. However, Tb.N in the posteroinferior region in *Homo* is higher than the other regions, overlapping with other taxa. Furthermore, *Pongo* has significantly lower Tb.N than *Pan* (*p* < 0.05) only in the posterosuperior region of the lateral condyle. In the medial condyle, *Gorilla* similarly show significantly lower Tb.N than *Pongo* and *Pan* in all regions (*p* < 0.01, and *p* < 0.001 respectively), but lower Tb.N than *Homo* only in the posteroinferior region (*p* < 0.01). *Pan* and *Pongo* again only differ in the posterosuperior region (*p* < 0.01), with *Pongo* having a lower Tb.N. *Pongo* has significantly higher Tb.N than *Homo* in the distal region (*p* < 0.05) and *Pan* shows significantly higher values than *Homo* in the distal (*p* < 0.05) and posterosuperior (*p* < 0.001) regions.

In the lateral condyle, Tb.Sp is significantly higher in *Gorilla* than in *Pan* and *Pongo* in all regions (*p* < 0.001 and *p* < 0.01 respectively; posterosuperior with *Pongo p* < 0.05). Moreover, Tb.Sp is higher than in *Homo* in the inferior regions (*p* < 0.01). *Pan* and *Homo* only differ in the posterosuperior region (*p* < 0.001), where *Pan* shows significantly lower Tb.Sp. No differences are found between *Pongo* and *Pan*, or *Pongo* and *Homo*. In the medial condyle, *Gorilla* again show significantly higher Tb.Sp in all regions than *Pan* and *Pongo* (*p* < 0.001 and *p* < 0.01 respectively), but only higher Tb.Sp in the posteroinferior region than *Homo* (*p* < 0.01). *Pongo* shows significantly higher Tb.Sp than *Pan* in the posterosuperior region (*p* < 0.05), but no significant differences to *Homo*, whereas *Homo* shows significantly higher Tb.Sp than *Pan* in the distal (*p* < 0.01) and posterosuperior (*p* < 0.001) regions. CV values show that in both condyles *Pan* is the most variable in the distal region, all species show similar variation in the posteroinferior region and *Homo* shows the greatest variation in the posteriosuperior region.

In regards to Tb.Th, in the lateral condyle, *Gorilla* shows significantly higher values than *Pan* in all regions (*p* < 0.01 and *p* < 0.001 in the posterosuperior). Furthermore, *Gorilla* has significantly higher Tb.Th than *Pongo* in the inferior regions (*p* < 0.05) and, when the captive specimens are removed, a significant difference is also detected in the posterosuperior region (*p* < 0.01). The only difference detected between *Gorilla* and *Homo* is in the posteroinferior region (*p* < 0.05), where *Gorilla* has higher Tb.Th. *Pan* shows significantly lower Tb.Th than *Homo* in the distal and posterosuperior regions (*p* < 0.05), whereas *Pongo* shows significantly lower Tb.Th than *Homo* only in the distal region of this condyle (*p* < 0.05). No significant differences are detected between *Pongo* and *Pan*. In the medial condyle, *Pan* displays significantly lower Tb.Th than *Gorilla* and *Homo* in all regions (*Gorilla p* < 0.001 and *p* < 0.01 in the distal; *Homo p* < 0.001 in distal, *p* < 0.05 in posteroinferior, *p* < 0.01 in posterosuperior), but no differences with *Pongo*. Moreover, *Gorilla* shows significantly higher Tb.Th than *Pongo* in the distal and posterosuperior regions (*p* < 0.05), and when the captive specimens are removed this is extended to the posteroinferior region (*p* < 0.01). No differences are found between *Gorilla* and *Homo* in any region. Similarly to the lateral condyle, *Pongo* and *Homo* only differ in the distal region (*p* < 0.01), with the former having lower thickness than the latter. When the captive specimens are not included, a significant result is also found in the posteroinferior region (*p* < 0.05). *Pongo* is consistently the most variable taxon across all regions of both condyles.

**Figure 10:**
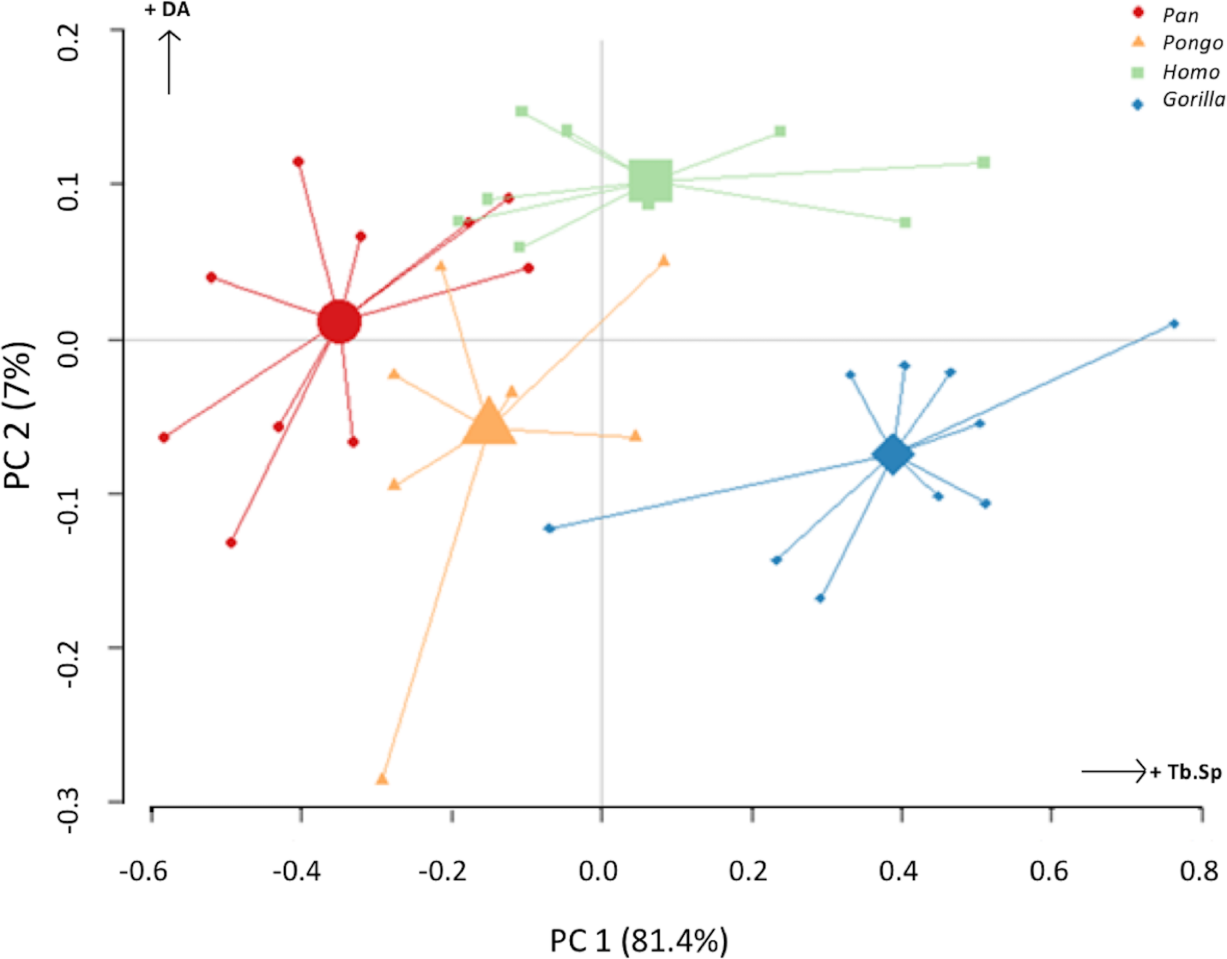
Results of principal components analysis of three trabecular variables (Tb.N, Tb.Sp. and DA) in all analysed regions. PC1 is mainly driven by variation in trabecular separation, while PC2 is driven primarily by degree of anisotropy (also see Table S2 for loadings).

The PC analysis of three trabecular variables (Tb.Th, Tb.Sp and DA) from all regions of both condyles reveals good separation among the different taxa (Fig. 10). Together, PC1 and PC2 explain 88% of the total variation (see Table S2 for loadings). The first PC separates *Gorilla*, with relatively high Tb.Sp, particularly in the medial condyle, from *Pan*, with relatively low Tb.Sp, while *Homo* and *Pongo* fall out as intermediate. The second PC primarily separates *Homo* with relatively high DA in both condyles from all other apes.

### Trabecular architecture and between-species regional relationships

**Figure 11:**
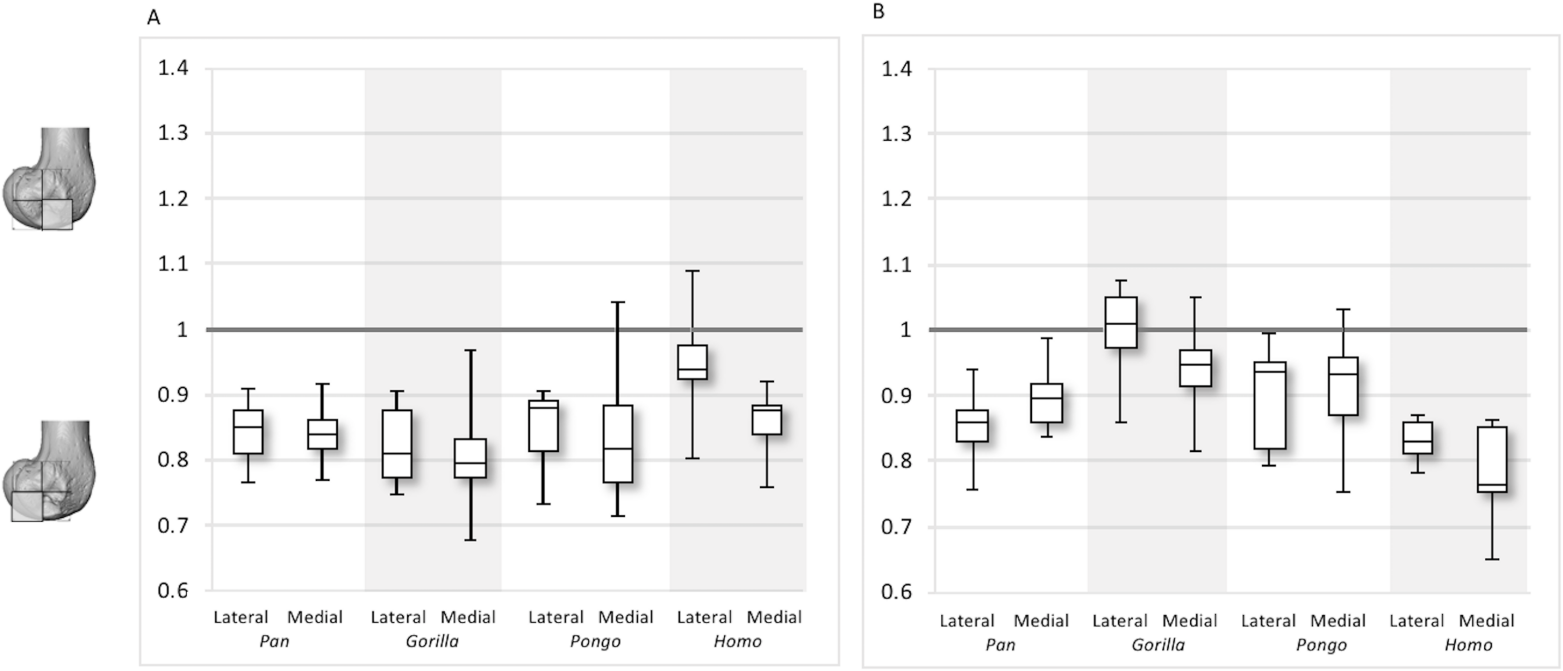
Inferior index for BV/TV and DA. (A) BV/TV. (B) DA. Index >1 indicates higher BV/TV or DA in the distal region, whereas index <1 indicates higher values in the posteroinferior region.

**Figure 12:**
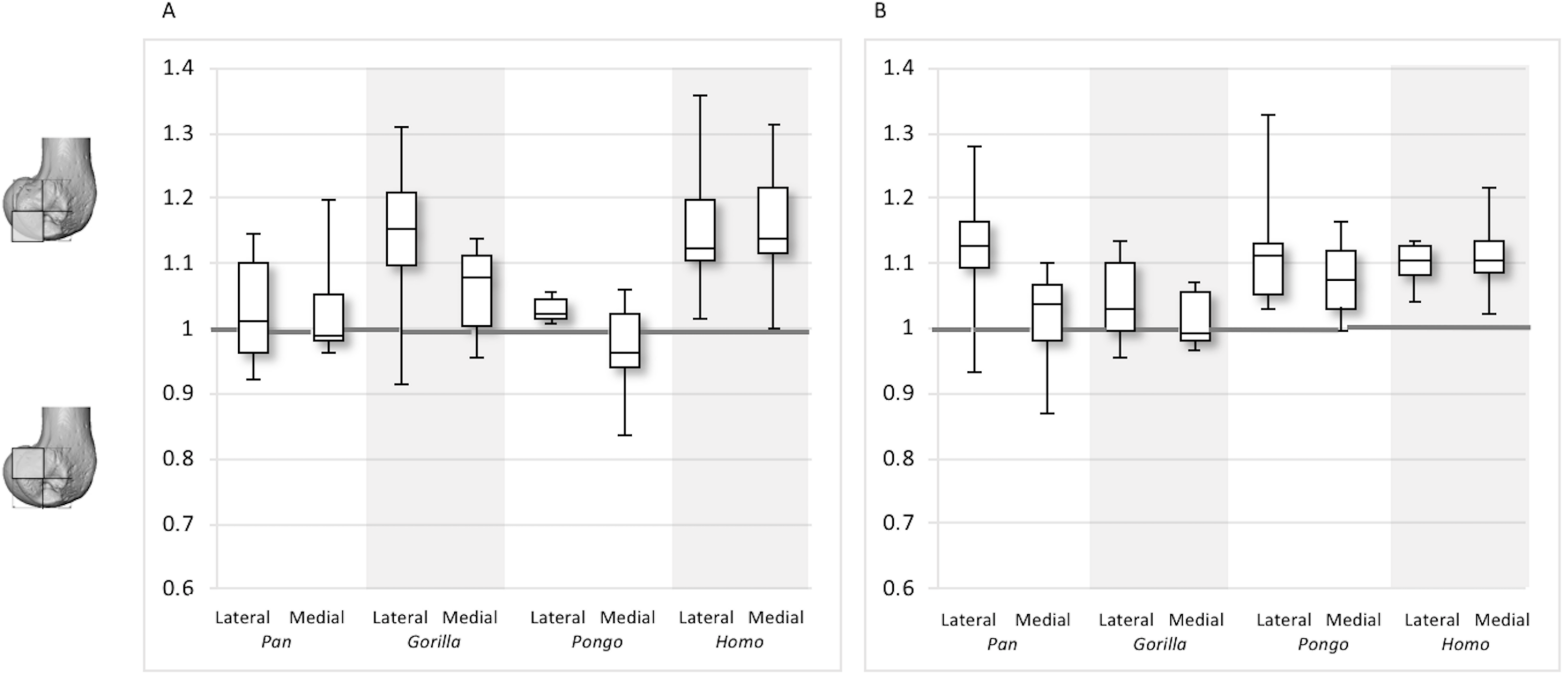
Posterior index for BV/TV and DA. (A) BV/TV. (B) DA. Index >1 indicates higher BV/TV or DA values in the posteroinferior region, whereas index <1 indicates higher values in the posterosuperior region.

Between-species variation is investigated further through two ratios that represent regional relationships in BV/TV and DA. The “inferior index” compares the distribution across the inferior regions of each condyle, where values >1 indicate higher BV/TV or DA in the distal versus the posteroinferior region. The “posterior index” compares distribution across posterior regions, where values >1 indicate higher BV/TV or DA in the posteroinferior versus the posterosuperior region. Results are displayed in Figs. 11–12 and detailed in Table 3 and Table S3. The BV/TV inferior index is <1 in all taxa and in both condyles, indicating that the posteroinferior region has consistently higher BV/TV than the distal region. However, in the lateral condyle, the *Homo* inferior index approaches 1 indicating that BV/TV is fairly equal across the inferior regions and it differs significantly from that of *Pan* (*p* < 0.05) and *Gorilla* (*p* < 0.01), but not *Pongo*. Thus, there is a greater disparity in BV/TV distribution between the inferior regions of the lateral condyle in African apes compared to humans. In the medial condyle no significant differences are found in the inferior index, indicating that the studied taxa have more similar relative distribution in BV/TV.

**Table 3:** Indices results for lateral and medial condyle.

Taxon	Parameter	Inferior lateral index	Posterior lateral index	Inferior medial index	Posterior medial index
*Pan*	BV/TV	0.84 (0.05)	1.03 (0.08)	0.84 (0.05)	1.03 (0.08)
	DA	0.86 (0.05)	1.13 (0.09)	0.90 (0.04)	1.02 (0.07)
*Gorilla*	BV/TV	0.86 (0.06)	1.15 (0.11)	0.81 (0.08)	1.06 (0.07)
	DA	1.00 (0.07)	1.04 (0.06)	0.94 (0.07)	1.01 (0.04)
*Pongo*	BV/TV	0.85 (0.06)	1.03 (0.02)	0.84 (0.11)	0.97 (0.08)
	DA	0.90 (0.08)	1.12 (0.10)	0.91 (0.09)	1.08 (0.06)
*Homo*	BV/TV	0.95 (0.08)	1.16 (0.11)	0.86 (0.05)	1.15 (0.10)
	DA	0.83 (0.03)	1.10 (0.04)	0.78 (0.07)	1.11 (0.05)

**Note:**

Mean values and standard deviation are provided for each index.

The inferior index also reveals interspecific differences in DA regional relationships. In the lateral condyle, *Homo* demonstrates the lowest ratio, indicating greater disparity in DA between the two inferior regions, with higher DA found in the posteroinferior region. In contrast, *Gorilla* has an inferior index approaching 1, indicating more equal DA across inferior regions. In lateral condyle, the inferior index differs significantly between *Gorilla* and *Homo* (*p* < 0.001), as well as *Gorilla* and *Pan* (*p* < 0.01). In the medial condyle, all taxa show a mean inferior index <1, indicating that the posteroinferior has relatively greater DA than the distal region. However, one *Pongo* specimen and two *Gorilla* specimens are >1. *Homo* displays the greatest disparity in DA between the two regions, with a significantly lower index than *Pan* (*p* < 0.01) and *Gorilla* (*p* < 0.001). All nonhuman apes are not significantly different from each other.

For the BV/TV posterior index in the lateral condyle, *Pan* and *Pongo* have a value close to 1 indicating a relatively equal distribution of BV/TV between the posteroinferior and posterosuperior regions. In contrast, both *Homo* and *Gorilla* show an index >1, indicating relatively higher BV/TV in the posteroinferior region. In the medial condyle, *Homo* shows the highest posterior index >1, indicating relatively higher BV/TV in the posteroinferior region, while the nonhuman apes show lower indices. *Pan* and *Pongo* show relatively equal values across the two regions with indices close to 1. The posterior index is significantly higher in *Homo* compared to *Pongo* in both condyles (lateral *p* < 0.05; medial *p* < 0.01) and compared to *Pan* in the medial condyle (*p* < 0.05) only. There are no significant differences between nonhuman apes.

For the DA posterior index in the lateral condyle, *Pan*, *Pongo* and *Homo* have indices >1, indicating relatively higher DA in the posteroinferior region compared with the posterosuperior. The *Gorilla* posterior index is closer to 1, indicated that DA is similar across the posterior regions of the lateral condyle. However, there are no significant differences in the DA indices across the taxa. In the medial condyle, *Pongo* and *Homo* show greater DA in the posteroinferior than the posterosuperior region, whereas *Gorilla* and *Pan* have indices closer to 1 indicating a relatively equal DA across these regions in African apes. Between-species comparisons of the index reveal that *Homo* has a significantly higher index than *Pan* (*p* < 0.05) and *Gorilla* (*p* < 0.001).

## Discussion

This study investigated trabecular variation in the distal femur of great apes and humans. We expected variation to reflect differences in locomotion and predicted differences in habitual joint posture, as well as habitual range of motion at the knee joint. We found general support for our predictions, although variation in BV/TV distribution did not clearly distinguish taxa despite (presumably) distinct differences in knee posture and loading during locomotion. We first discuss intraspecific variation, followed by interspecific differences.

### Within-species trabecular patterns

The *Pan* distal femur had particularly high BV/TV in the posterosuperior and posteroinferior regions of both condyles, and comparatively low BV/TV in the distal region. Higher BV/TV values extended from the subchondral surface relatively far into the epiphysis of both condyles, particularly in the medial condyle (Fig. 4). Quantification of the trabecular architectural variables revealed that the high BV/TV in *Pan* was characterised by numerous, thin trabeculae with narrow separation. Furthermore, DA was highest in the posteroinferior region in the lateral condyle, but equally low in the two other regions. In the medial condyle DA is more equal across posterior regions, but low in the distal region. Together, these results are consistent with higher and more uniaxial loading of the distal femur in a flexed-knee posture, which is used during both quadrupedal knuckle-walking and, especially, vertical climbing (D’Août et al., 2002, 2004; Isler, 2005). The more isotropic posterosuperior region may reflect the more variable loading that would occur during climbing, as this region is (presumably) in contact with the proximal tibia only when the knee is strongly flexed (Isler, 2005; Fig. 1).

*Gorilla* showed high BV/TV in the posteroinferior region, which did not always extend posterosuperiorly. The disparity between BV/TV in the posterior regions was more obvious in the lateral condyle, where the BV/TV of the posteroinferior region was visibly higher. In the medial condyle, BV/TV values were similar across the posterior regions. In both condyles BV/TV was lowest in the distal region, where trabecular separation was highest, perhaps consistent with decreased loading of this region. The BV/TV concentration did not extend far within the epiphysis. In both condyles, there was a similar DA across the three studied regions; however, DA in the medial condyle was generally higher than that of the lateral condyle, perhaps due to the greater loading experienced by this condyle (Preuschoft & Tardieu, 1996). Moreover, *Gorilla* displayed fewer but thicker and more widely-separated trabeculae than the other taxa in all of the analysed regions, suggesting that increasing the thickness of trabeculae is important in mitigating load.

The trabecular structure of the *Pongo* distal femur was the most variable across the sample. In general, BV/TV was lowest in the distal region of both condyles. In the lateral condyle BV/TV was highest in the posteroinferior region. However, in the medial condyle some individuals showed higher BV/TV values in the posterosuperior region while other showed fairly equal values across both posterior regions. The great range of values in all studied regions revealed high intraspecific variation in the distribution of BV/TV within the condyles. The high BV/TV was characterised by numerous trabeculae that were relatively thin and closely packed in all regions. *Pongo* showed relatively low DA values across all regions of the epiphysis, particularly in the medial condyle. Together, these results are consistent with the highly mobile knee joint (Morbeck & Zihlman, 1988; Tuttle & Cortright, 1988) that facilitates more variable loading of the distal femur during a diverse arboreal locomotor repertoire (Cant, 1987; Thorpe & Crompton, 2006; Thorpe, Holder & Crompton, 2007, 2009). Notably, most *Pongo* specimens had a concentration of high BV/TV at the posterior shaft just superior to the femoral condyles. This region underlies the insertion site for the heads of the gastrocnemious muscle (Prejzner-Morawska & Urbanowicz, 1981; Diogo et al., 2010, 2013a, 2013b). This could be the result of the gastrocnemius muscle being strongly recruited during suspension by the hindlimbs, which is more frequently practiced in *Pongo* than in African apes (Thorpe & Crompton, 2006). However, the gastrocnemius is recruited during bipedal walking and running in humans (Neptune, Kautz & Zajac, 2001; Ishikawa, Pakaslahti & Komi, 2007; Lichtwark, Bougoulias & Wilson, 2007), and is presumably also important during knuckle-walking and climbing in African apes.

The comparatively high degree of variability within *Pongo* is not necessarily surprising. Distal femur posture and loading during locomotion can vary between species (Mackinnon, 1974; Manduell, Harrison & Thorpe, 2012) and between individuals due to differences in sex and/or body size (Sugardjito & van Hooff, 1986; Cant, 1987; Thorpe & Crompton, 2005). *Pongo* was the only sample in our study to comprise two species (*P. abelii* and *P. pygmaeus*), although there were no consistent differences in trabecular structure found between these species in our small sample. Furthermore, our sample also included two captive specimens; one female (*Pongo sp.*) and the other being the only male (*P. pygmaeus*) in the sample. These individuals regularly fell out as outliers in the *Pongo* sample for BV/TV, DA and Tb.Th, even though interspecific differences were not largely affected. Both showed higher BV/TV and Tb.Th than the other *Pongo* specimens in most regions, which is perhaps explained by their altered locomotion in captivity. Isler & Thorpe (2003) found that captive *Pongo* used shorter gait cycles and faster speed then wild individuals, likely because the captive environment was more predictable. Furthermore, the captive male *Pongo* specimen consistently showed the highest DA values in the sample, coupled with the lowest trabecular number in most regions, while the female displayed the lowest DA values. The trabecular architecture of the male is in line with less climbing behaviour and reflects an altered response to load in larger-sized individuals, whereas that of the female may be a result of more variable and arboreal behaviours resulting in more isotropic trabecular structure. Nonetheless, Tb.N and Tb.Sp mostly fall within the range of wild shot *Pongo* individuals. Given the limited number of *Pongo* specimens available in osteological collections, a fruitful avenue of future research would be to systematically compare trabecular structure between wild and captive specimens, particularly if general activity patterns are known in the latter.

*Homo* showed highest BV/TV in the posteroinferior region. The posterosuperior region showed consistently lower values but as BV/TV in the distal region was more variable, patterns between the condyles differed. In the lateral condyle values in the distal region were generally high compared to those of the medial condyle and were higher than the values in the posterosuperior region; a pattern opposite to what is found in the medial condyle. The DA values were greatest in the posteroinferior region and lowest in the distal region of both condyles. High BV/TV in the posteroinferior region of both condyles was characterised by more numerous trabeculae that were more closely packed but less thick compared with the other regions of the *Homo* distal femur. This trabecular pattern is consistent with the region of highest loading when GRFs (Racic, Pavic & Brownjohn, 2009) and joint reaction forces (Nordin & Frankel, 2001) are highest during the gait cycle, right before toe-off. The absence of high bone concentration in the posterosuperior region of both condyles is consistent with the relative infrequency of using a highly-flexed knee posture during habitual activities. However, the relatively high intraspecific variation in BV/TV distribution within the *Homo* sample, indicated by generally higher CV values than African apes, was somewhat surprising. Despite humans loading their knees in stereotypical ways compared with other apes, this could be the result of frequent use of behaviours not considered in the predictions of this study, including climbing stairs, sitting, squatting or running, all of which result in different flexion angles (Hardt, 1978; Baltzopoulos, 1995; Simpson & Pettit, 1997; Zheng et al., 1998; Anderson & Pandy, 2001; Kellis, 2001; Nagura et al., 2002; Taylor et al., 2004). Changes in knee angle have been shown to affect joint reaction force and contact area. For example, more flexed knee postures result in higher forces on the articular surface (Taylor et al., 2004; Kutzner et al., 2010) and a larger contact area at the posterior end of the condyles (von Eisenhart-Rothe et al., 2004). In contrast, more extended knee postures result in a smaller contact area that is more centrally located on the condyles. Unfortunately, the lack of additional life-history information on the human sample deems this speculative. Alternatively, this could be due to a lack of a clear functional signal in the trabecular structure of the human distal femur.

### Between-species trabecular differences

Our results revealed several interspecific differences in the trabecular structure of the distal femur across hominoids, although these differences were less pronounced than we predicted. We predicted that *Homo* would have absolutely lower BV/TV values compared with great apes and that the BV/TV distribution would be distally concentrated in the condyles reflecting a habitually extended knee posture. This prediction was not fully supported. *Homo* did not have significantly lower BV/TV in the studied regions compared to great apes, which is in contrast to recent findings that more sedentary recent humans have systemically lower BV/TV throughout various regions of the skeleton (Chirchir et al., 2015, 2017; Ryan & Shaw, 2015; Saers et al., 2016). However, our results are in line with recent findings that humans do not consistently display significantly lower BV/TV than *Pan* across skeletal sites (Tsegai et al., 2018). Unfortunately, as we do not have information on the activity levels or professions of the human population in this study, it is difficult to interpret this result. Nonetheless, the high BV/TV values of the inferior regions and the lack of this BV/TV concentration posterosuperiorly is consistent with extended-knee locomotion.

We predicted that *Pan* and *Gorilla* would show similar, high BV/TV concentrations posterosuperiorly, reflecting the use of more flexed positions. This prediction was supported by the greater BV/TV in the posteroinferior compared to the distal region in both taxa and the high BV/TV in the posterosuperior region in *Pan* consistent with loading of the condyles in more flexed postures. *Pan* showed greater BV/TV concentration in the posterior regions than *Homo*, supporting our prediction, but differed from the pattern found in *Gorilla*. The lack of the posterosuperior concentration in *Gorilla* is consistent with their more extended-knee posture during terrestrial locomotion (Hofstetter & Niemitz, 1998; Isler, 2005; Crompton, Vereecke & Thorpe, 2008; but see Finestone et al., 2018), less flexion at the knee during climbing (Isler, 2002, 2005) and a locomotor repertoire that includes more frequent knuckle-walking and less climbing compared with *Pan* (Tuttle & Watts, 1985; Crompton, Sellers & Thorpe, 2010).

We also predicted that *Pongo* would show homogenous BV/TV distribution across all analysed regions of the distal femur, reflecting more variable knee joint loading. Our results suggest that the distribution is not homogenous in *Pongo* and the pattern does not differ significantly to that of *Pan*. *Pan* and *Pongo* showed high BV/TV values across the posterior regions, consistent with the frequent adoption of both flexed and hyperflexed joint positions consistent with quadrupedal terrestrial locomotion and vertical climbing respectively. The high degree of intraspecific variability found in *Pongo* is consistent with previous comparative trabecular studies on other skeletal elements (Schilling et al., 2014; Tsegai et al., 2013) and thus further investigation into the factors, including genetic, development, hormonal or biomechanical factors, influencing this intraspecific variability is needed.

Furthermore, we predicted that within our sample, *Homo* would show the highest DA throughout the distal femur reflecting the stereotypical loading that occurs during habitual bipedalism, while *Pan* and *Gorilla* would show similar intermediate levels of DA, and that *Pongo* would show the lowest DA values. Our predictions were generally supported. *Homo* had comparatively higher DA in all regions of the distal femur compared with other great apes and the overall pattern was distinctly different from what was found in African apes and *Pongo*. These differences could be explained by variation in mediolateral motion between taxa and less variability in joint forces during locomotion in *Homo* (Preuschoft & Tardieu, 1996). Femoral movement within the tibio-femoral joint is the result of both hard and soft tissue morphology (Reynolds, Walker & Buza, 2017). Both cruciate ligaments prevent tibial displacement (Butler, Noyes & Grood, 1980), whereas the collateral ligaments stop valgus or varus rotation (Shoemaker & Markolf, 1985; Gollehon, Torzilli & Warren, 1987). The quadriceps, gastrocnemius and hamstrings also assist with knee stability (Shelburne, Torry & Pandy, 2006). “Independent rotation” is dictated by the fit with the tibia, which varies across hominoids. In *Homo*, the width of the intercondyloid notch is similar to that of the tibial interspinal distance (Tardieu, 1981), resulting in more constriction of movement and limited independent rotation of the two elements. In the rest of the great apes this trait varies with body size (Tardieu, 1981). *Pan* has the greatest disparity in fit, followed by *Pongo* and then *Gorilla*, displaying differences in knee rotational capacity. Furthermore, the larger articular surface of the medial condyle than that of the lateral in nonhuman apes (Tardieu, 1981) assists in ‘combined rotation’, where rotation and flexion–extension happen simultaneously. This external rotation during extension is evident in *Pongo* and *Pan* (Lovejoy, 2007). Greater rotation in these taxa suggests that resulting forces are multi-axial, loading the knee in several directions and therefore producing less anisotropic trabecular structure within the condyles. In contrast, the *Homo* knee is more restricted and, even when flexing, there is a lack of significant mediolateral rotation. This results in more uniform loading and, consequently, a higher degree of trabecular anisotropy.

Lastly, we predicted that trabecular architectural variables would reflect differences in body size consistent with previous studies (Doube et al., 2011; Ryan & Shaw, 2013; Barak, Lieberman & Hublin, 2013). Specifically, we predicted that smaller-bodied *Pan* and *Pongo* would show higher Tb.N but lower Tb.Sp and Tb.Th, while larger-bodied *Homo* and *Gorilla* would show the opposite pattern. Although we did not directly test allometry due to the small and unbalanced sex samples within each taxon, we found some support that trabeculae of the distal femur show a similar relationship with body size as found in previous studies. The smaller-sized taxa *Pongo* and *Pan* generally showed greater Tb.N and lower Tb.Sp and Tb.Th than the other hominoids. Conversely, the larger-sized *Gorilla* generally showed greater Tb.Th and Tb.Sp, but lower Tb.N than the other taxa. These results perhaps reveal a link between certain trabecular parameters and body size that could stem from differences during the modelling process. However, further investigation of potential allometric influence on trabecular structure within each taxon is needed on larger and more balanced-sex samples.

Although we found some clear differences in trabecular structure that are consistent with our predictions based on the knee joint range of motion and loading during habitual locomotion, the trabecular patterns revealed here are not necessarily straightforward. There was much greater overlap between *Homo* and other great apes than expected given their dramatic differences in knee joint posture and loading. Biomechanical inferences from trabecular structure are complex because it is not clear what triggers modelling or how trabecular and cortical bone respond to strain (Wallace et al., 2014); for example, research suggests that bone responds to high frequency, low intensity loading and low frequency, high intensity loading, as well as a range of loads that fall between the two extremes (Whalen, Carter & Steele, 1988; Rubin, McLeod & Bain, 1990; Rubin et al., 2001; Judex et al., 2003; Scherf, Harvati & Hublin, 2013). Additionally, we do not know if this differs between specialist and generalist species. Furthermore, it is difficult to control for factors such as genetics, age, hormones, demands for maintaining bone homeostasis and other systemic factors that could influence the organisation of trabecular bone (Simkin, Ayalon & Leichter, 1987; Lee et al., 2003; Pearson & Lieberman, 2004; Suuriniemi et al., 2004; Kivell, 2016; Wallace, Demes & Judex, 2017; Tsegai et al., 2018). It has been shown that bone mineral density, as well as bone turnover are to a great extent hereditary (Smith et al., 1973; Dequeker et al., 1987; Kelly et al., 1991; Garnero et al., 1996; Harris et al., 1998). Additionally, trabecular architecture across the skeleton is regulated by different genes (Judex et al., 2004), which adds to the complexity and extrapolating from one skeletal site to another may introduce error. Genotypic variations may also influence the response to mechanical strain (Judex, Donahue & Rubin, 2002), complicating functional interpretations even further. Thus, variation in bone’s response to different types of loading across skeletal sites, between sexes or pathological states (Goldstein, 1987; Keaveny et al., 2001; Yeni et al., 2011), as well as the influence of non-mechanical factors suggest that the study of this tissue is complex. Hence, there is a need to understand in greater depth how the knee joint functions and how load is distributed in the different regions of the condyles across hominoids so that we can better link variation in trabecular structure to mechanical loading, particularly in extinct taxa.

## Conclusion

This study provided the first holistic study of trabecular bone within the hominoid distal femur. We showed that humans, despite not being as distinct as initially predicted, are characterised by higher DA than of all other hominoids and more distally concentrated BV/TV compared with *Pan* and *Pongo*, which is consistent with more stereotypical loading in an extended-knee posture during bipedalism. *Pan* and *Pongo* showed more posteriorly-concentrated BV/TV and all apes show lower DA than humans; traits that are generally consistent with more variable loading in a flexed-knee posture that is used during knuckle-walking and climbing. Variation found in this study and specifically in *Pongo*, was consistent with the limited biomechanical studies of knee posture and loading, but substantial overlap in different trabecular parameters across taxa suggest caution is needed when making inferences about behaviour in fossil taxa.

## Supplemental Information

10.7717/peerj.5156/supp-1 Supplemental Information 1 Fig. S1. *Pan* BV/TV and DA distributions in the complete sample.

10.7717/peerj.5156/supp-2 Supplemental Information 2 Fig. S2. *Gorilla* BV/TV and DA distributions in the complete sample.

10.7717/peerj.5156/supp-3 Supplemental Information 3 Fig. S3. *Pongo* BV/TV and DA distributions in the complete sample. Captive specimens: ZSM 1966 0203 (male) and ZSM 1982 0092 (female).

10.7717/peerj.5156/supp-4 Supplemental Information 4 Fig. S4. *Homo* BV/TV and DA distributions in the complete sample.

10.7717/peerj.5156/supp-5 Supplemental Information 5 Table S1. Results (*p*-value) for between taxa differences in the examined regions. Captive *Pongo* are included.

10.7717/peerj.5156/supp-6 Supplemental Information 6 Table S2. Loadings of parameters at each region to PC1 and PC2.

10.7717/peerj.5156/supp-7 Supplemental Information 7 Table S3. Results (*p*-value) for between taxa differences in the indices. Captive *Pongo* are included.

10.7717/peerj.5156/supp-8 Supplemental Information 8 Trabecular parameters for each specimen.

## Additional Information and Declarations

### Data Availability

The following information was supplied regarding data availability: The raw data are provided in a Supplemental File.
