# Supplementary material for: Trabecular bone patterning in the hominoid distal femur
Source: PeerJ. 2018 Jul 5;6:e5156. doi: 10.7717/peerj.5156 (PMC6035864; doi:10.7717/peerj.5156)

Pan troglodytes verus BV/TV distribution

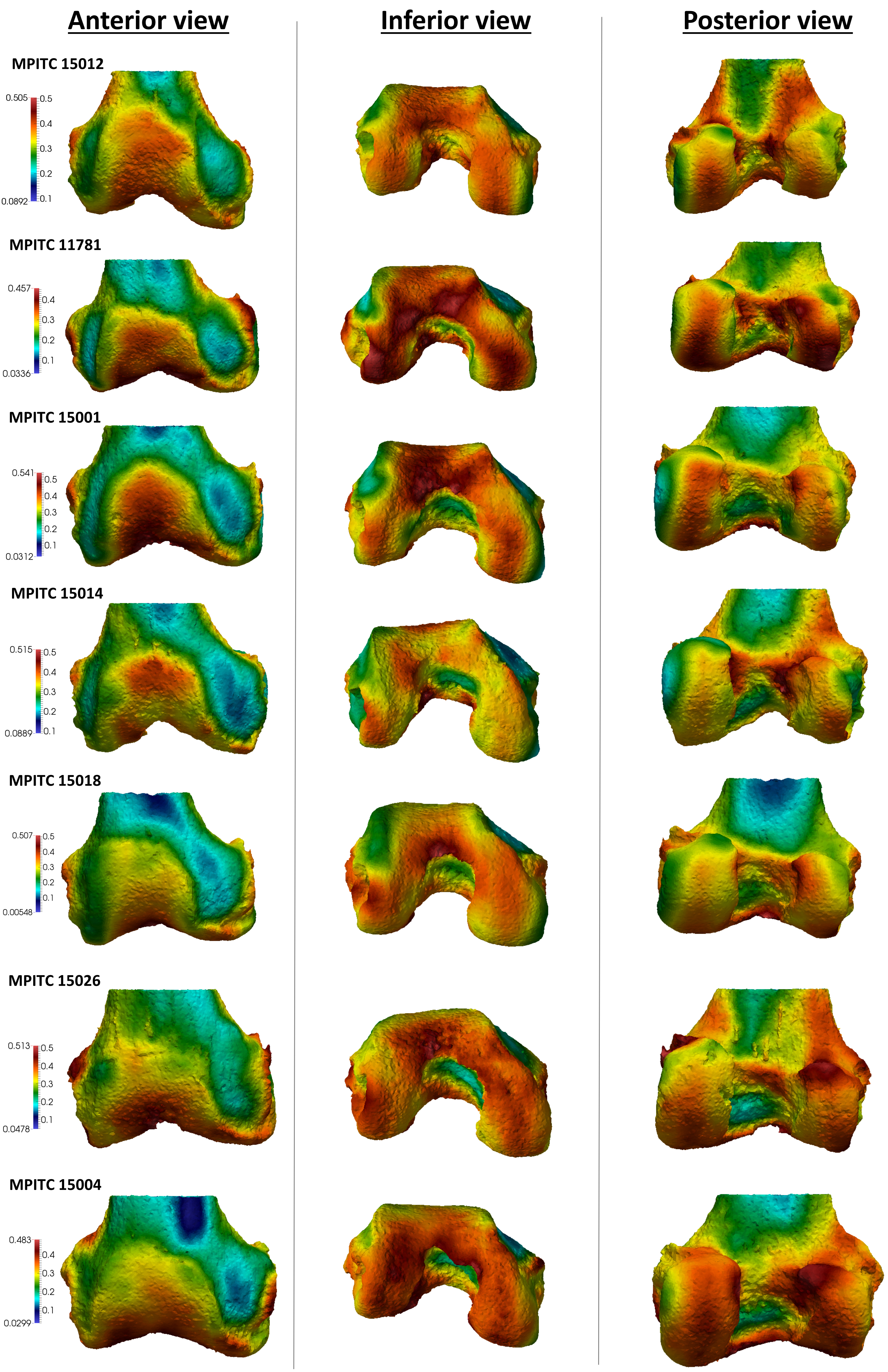

Pan troglodytes verus BV/TV distribution

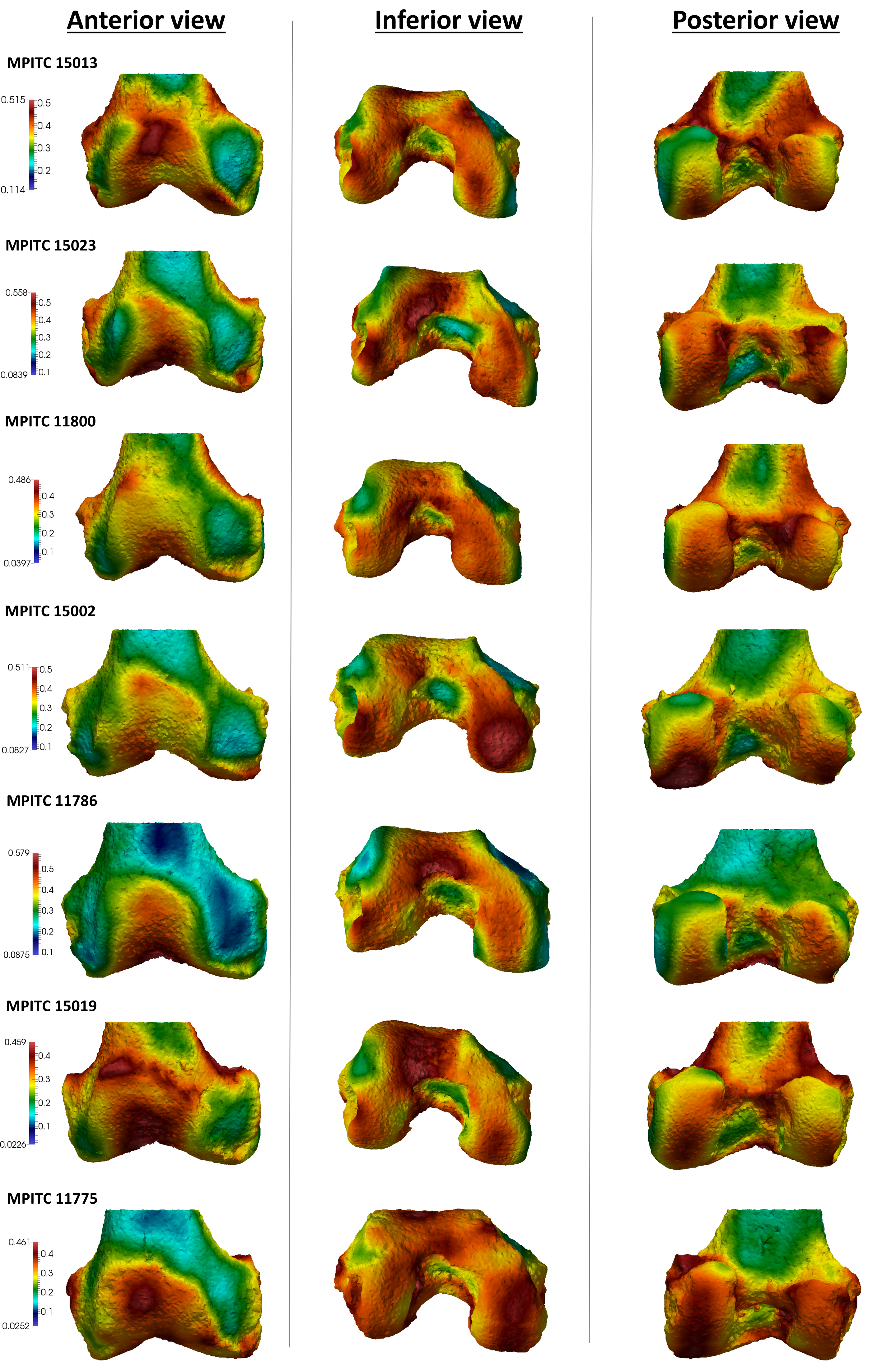

Pan troglodytes verus BV/TV distribution

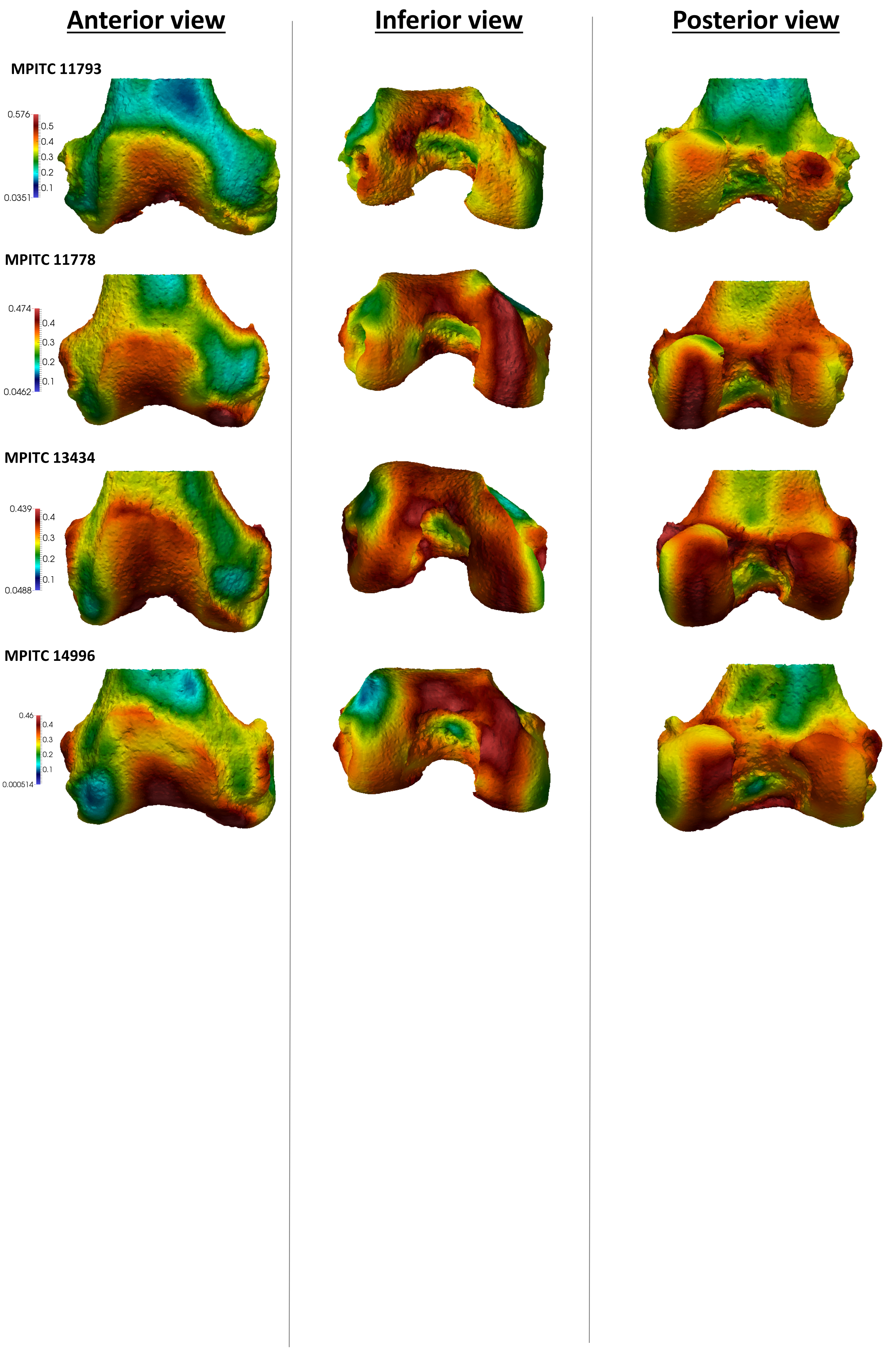

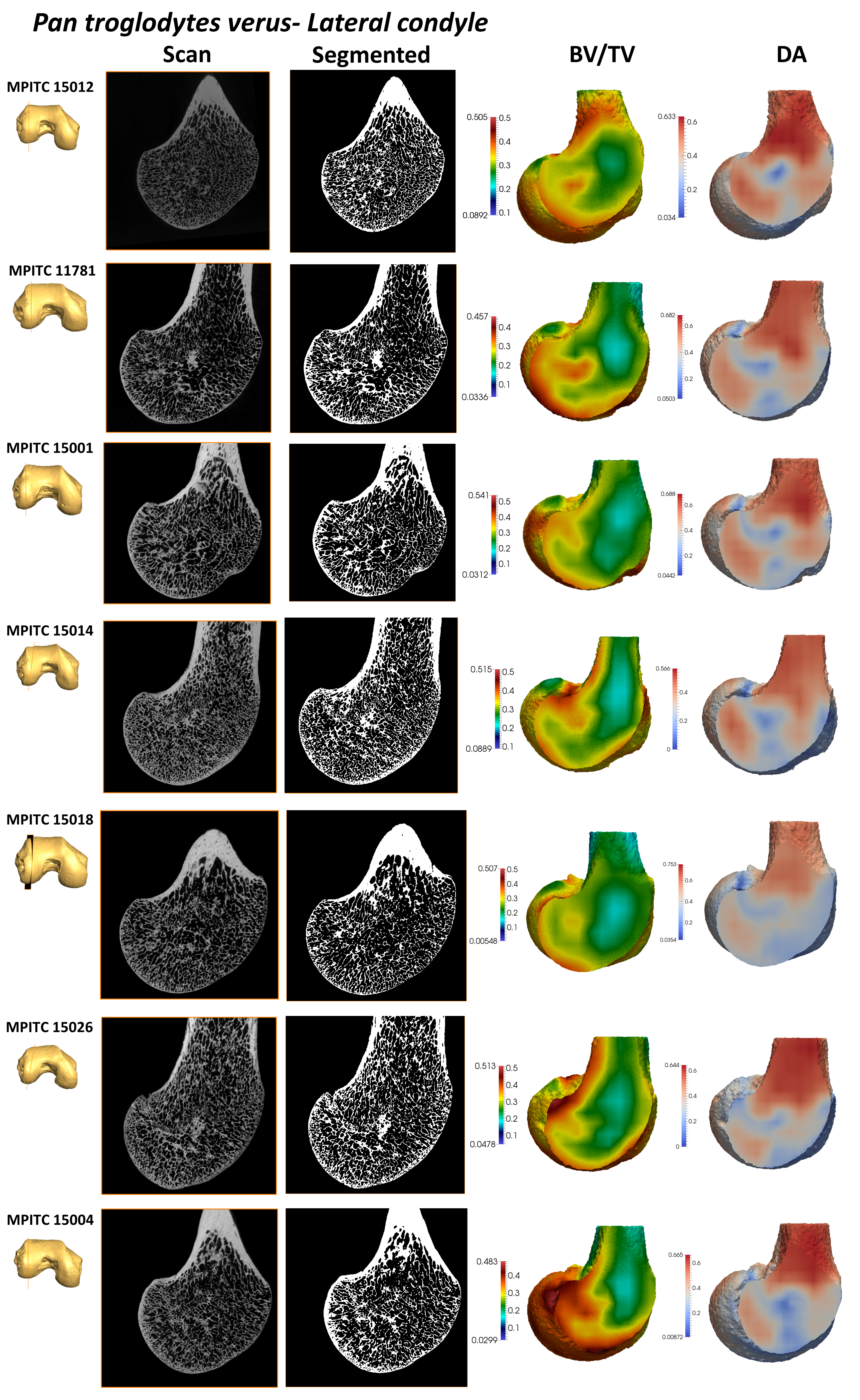

Pan troglodytes verus- Lateral condyle
Scan
Segmented
BV/TV
DA
MPITC 15012
MPITC 11781
MPITC 15001
MPITC 15014
MPITC 15018
MPITC 15026
MPITC 15004
0.505
0.0892
0.633
0.034
0.457
0.0336
0.682
0.0503
0.541
0.0312
0.688
0.0442
0.515
0.0889
0.566
0
0.507
0.00548
0.753
0.0354
0.513
0.0478
0.644
0
0.483
0.0299
0.665
0.00872

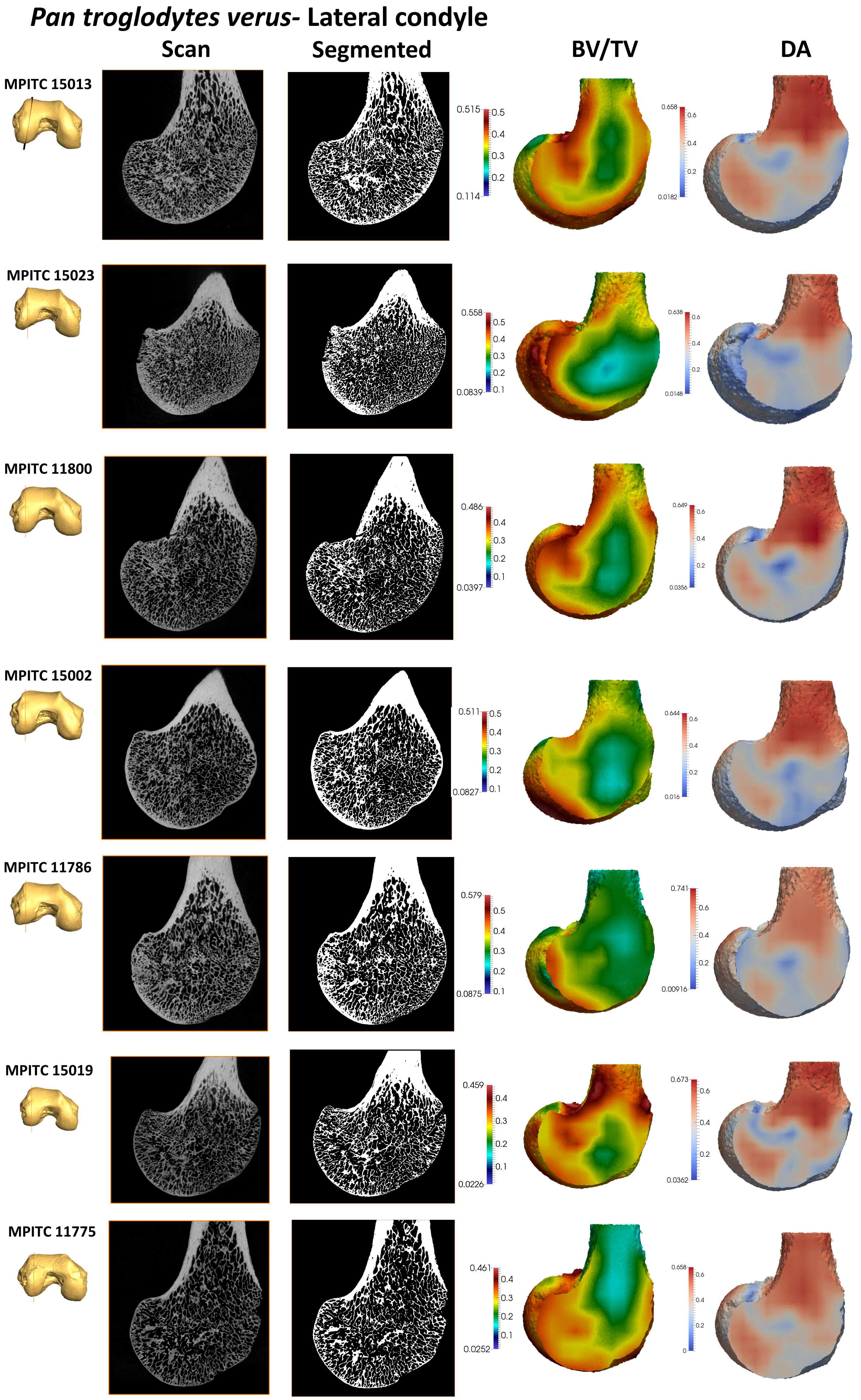

Pan troglodytes verus- Lateral condyle
Scan
Segmented
BV/TV
DA
MPITC 15013
MPITC 15023
MPITC 11800
MPITC 15002
MPITC 11786
MPITC 15019
MPITC 11775

Pan troglodytes verus- Lateral condyle

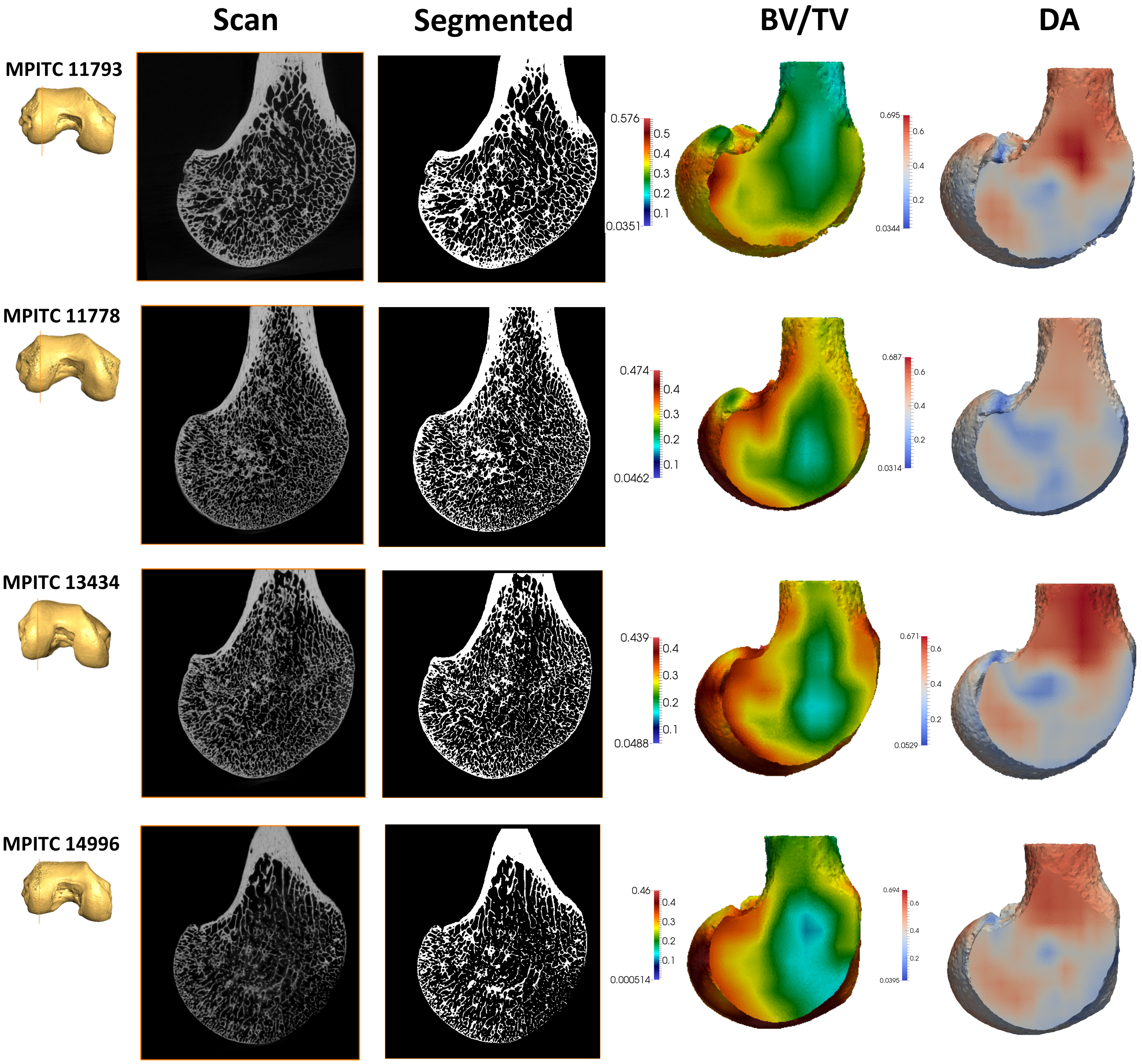

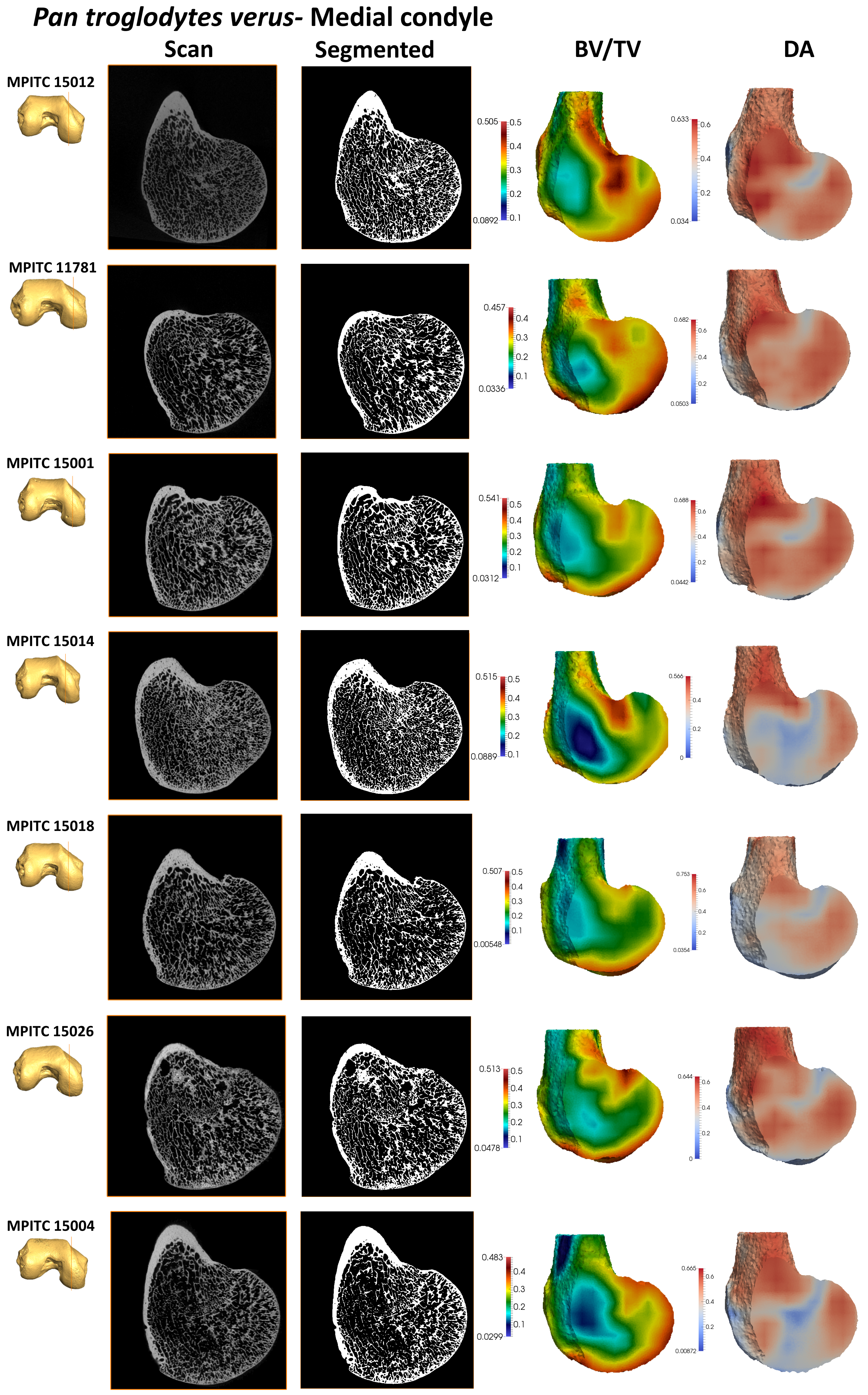
Pan troglodytes verus- Medial condyle
Scan
Segmented
BV/TV
DA
MPITC 15012
MPITC 11781
MPITC 15001
MPITC 15014
MPITC 15018
MPITC 15026
MPITC 15004
0.505
0.0892
0.633
0.034
0.457
0.0336
0.682
0.0503
0.541
0.0312
0.688
0.0442
0.515
0.0889
0.566
0.507
0.00548
0.753
0.0354
0.513
0.0478
0.644
0.483
0.0299
0.665
0.00872

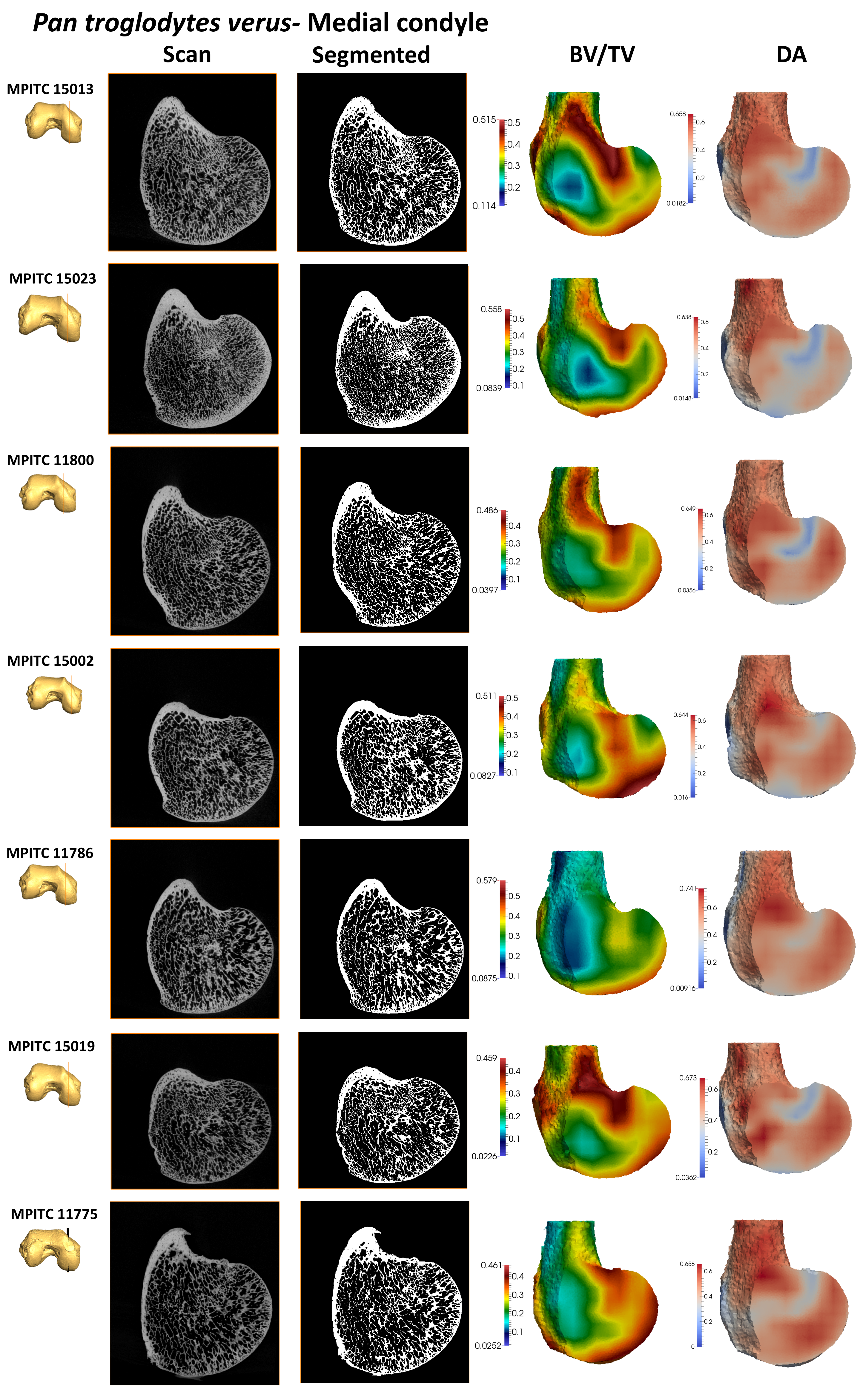
Pan troglodytes verus- Medial condyle
Scan
Segmented
BV/TV
DA
MPITC 15013
0.515
0.114
0.658
0.0182
MPITC 15023
0.558
0.0839
0.638
0.0148
MPITC 11800
0.486
0.0397
0.649
0.0356
MPITC 15002
0.511
0.0827
0.644
0.016
MPITC 11786
0.579
0.0875
0.741
0.00916
MPITC 15019
0.459
0.0226
0.673
0.0362
MPITC 11775
0.461
0.0252
0.658
0

Pan troglodytes verus- Medial condyle

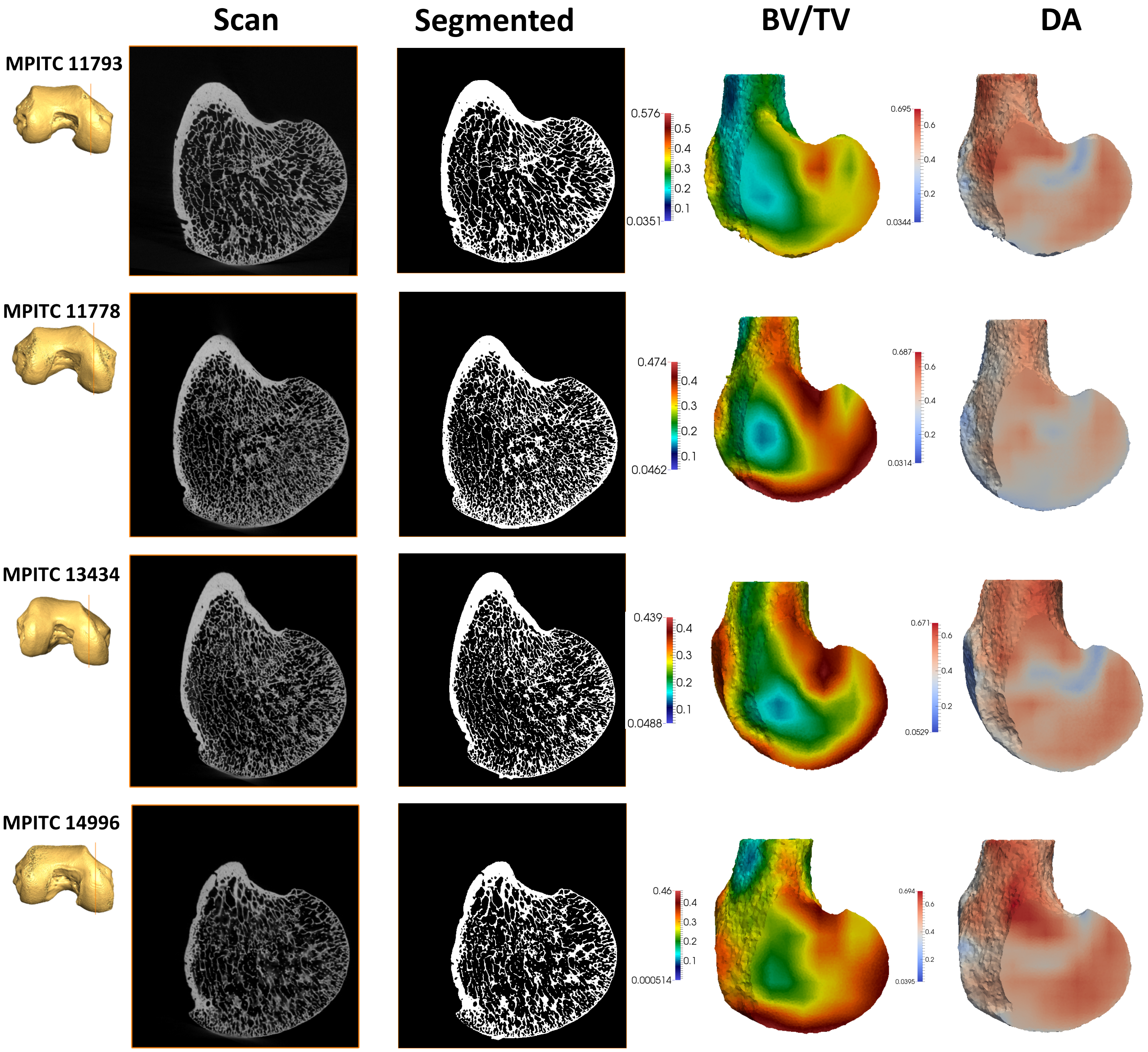

Supplement: Supplemental Information 1 [file peerj-06-5156-s001.pdf]
