# Supplementary material for: Trabecular bone patterning in the hominoid distal femur
Source: PeerJ. 2018 Jul 5;6:e5156. doi: 10.7717/peerj.5156 (PMC6035864; doi:10.7717/peerj.5156)
Supplement: Supplemental Information 2 [file peerj-06-5156-s002.pdf]

Gorilla gorilla gorilla- BV/TV distribution

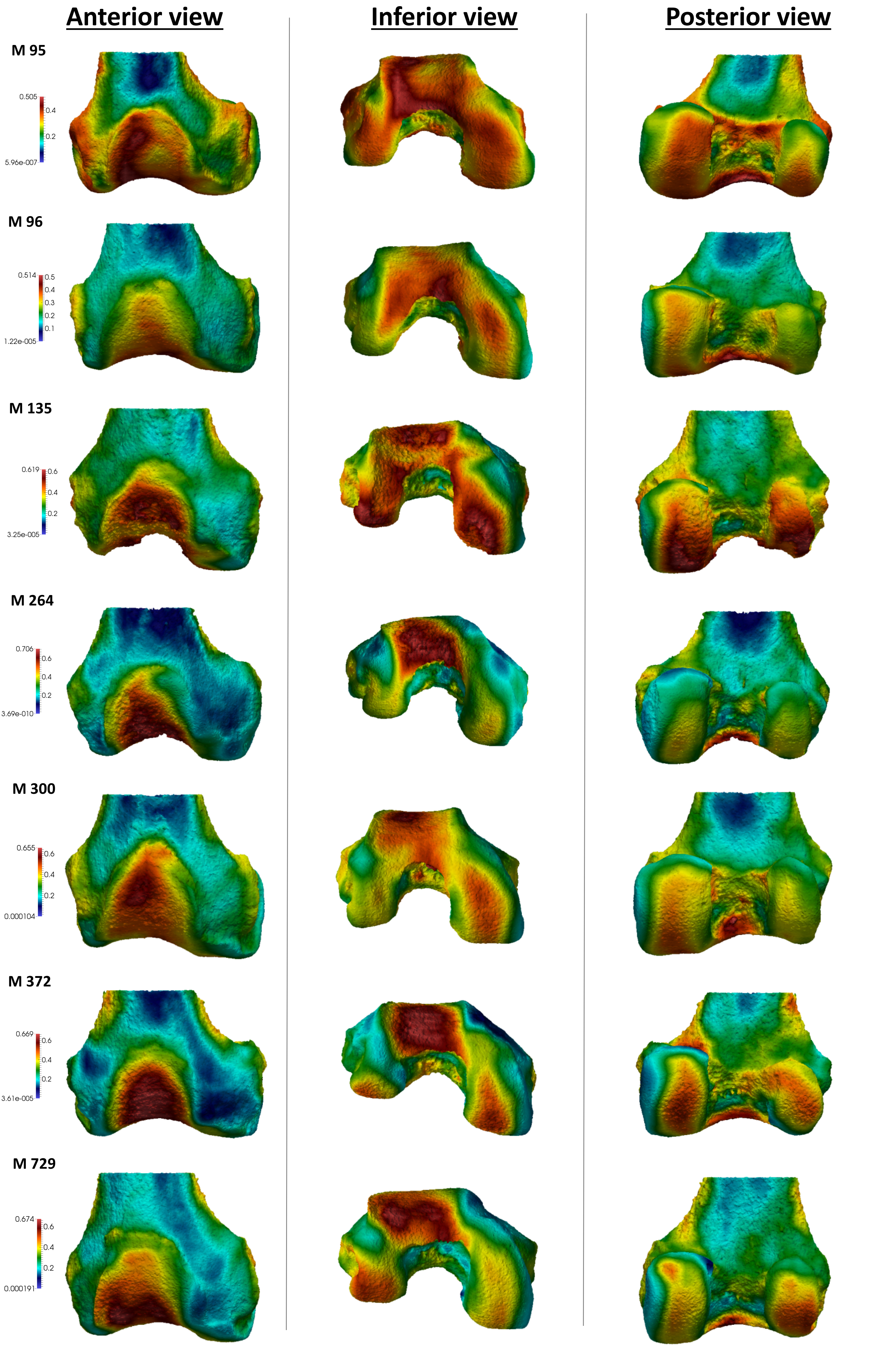

Gorilla gorilla gorilla- BV/TV distribution

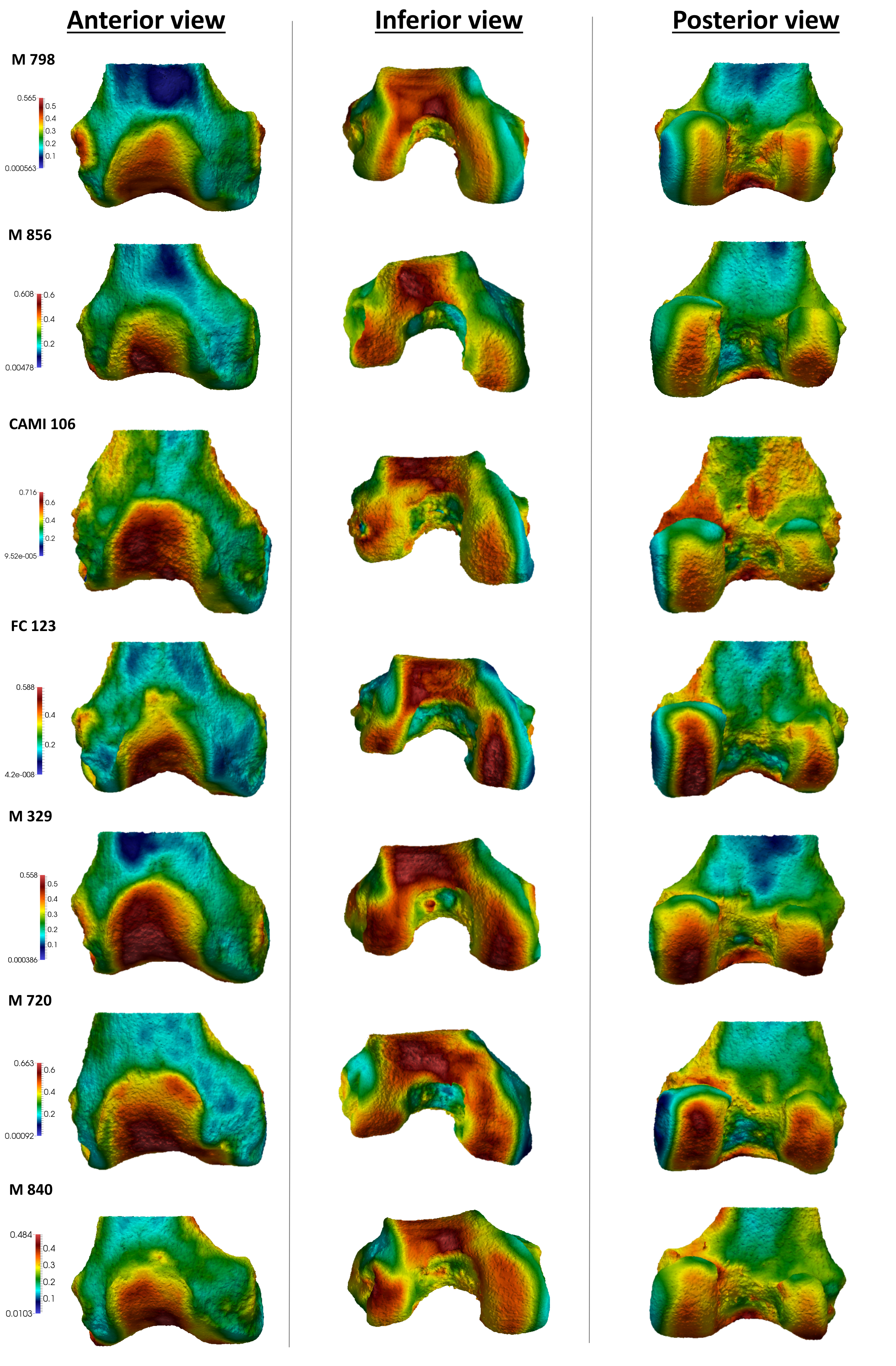

Gorilla gorilla gorilla- Lateral condyle

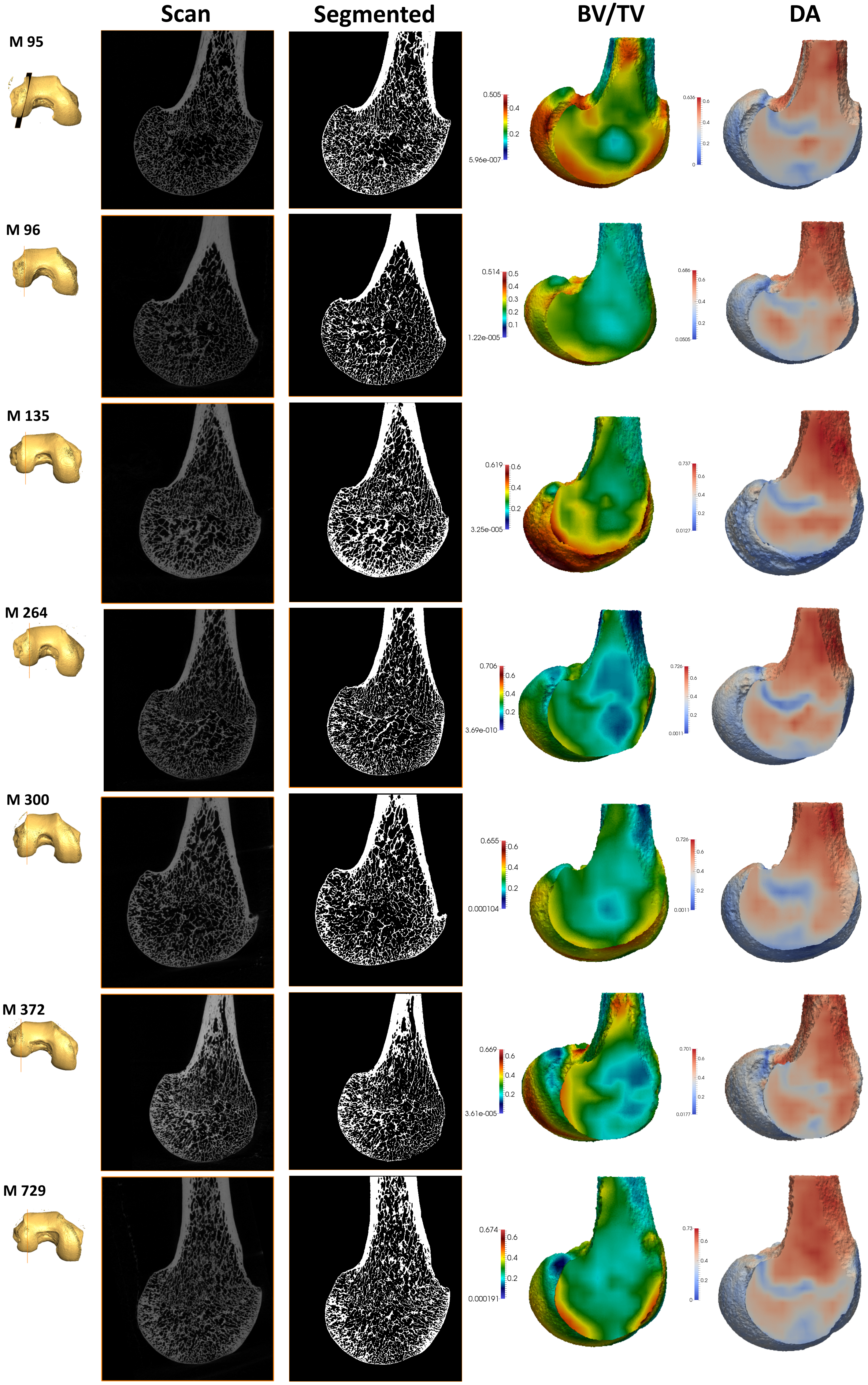

Gorilla gorilla gorilla- Lateral condyle

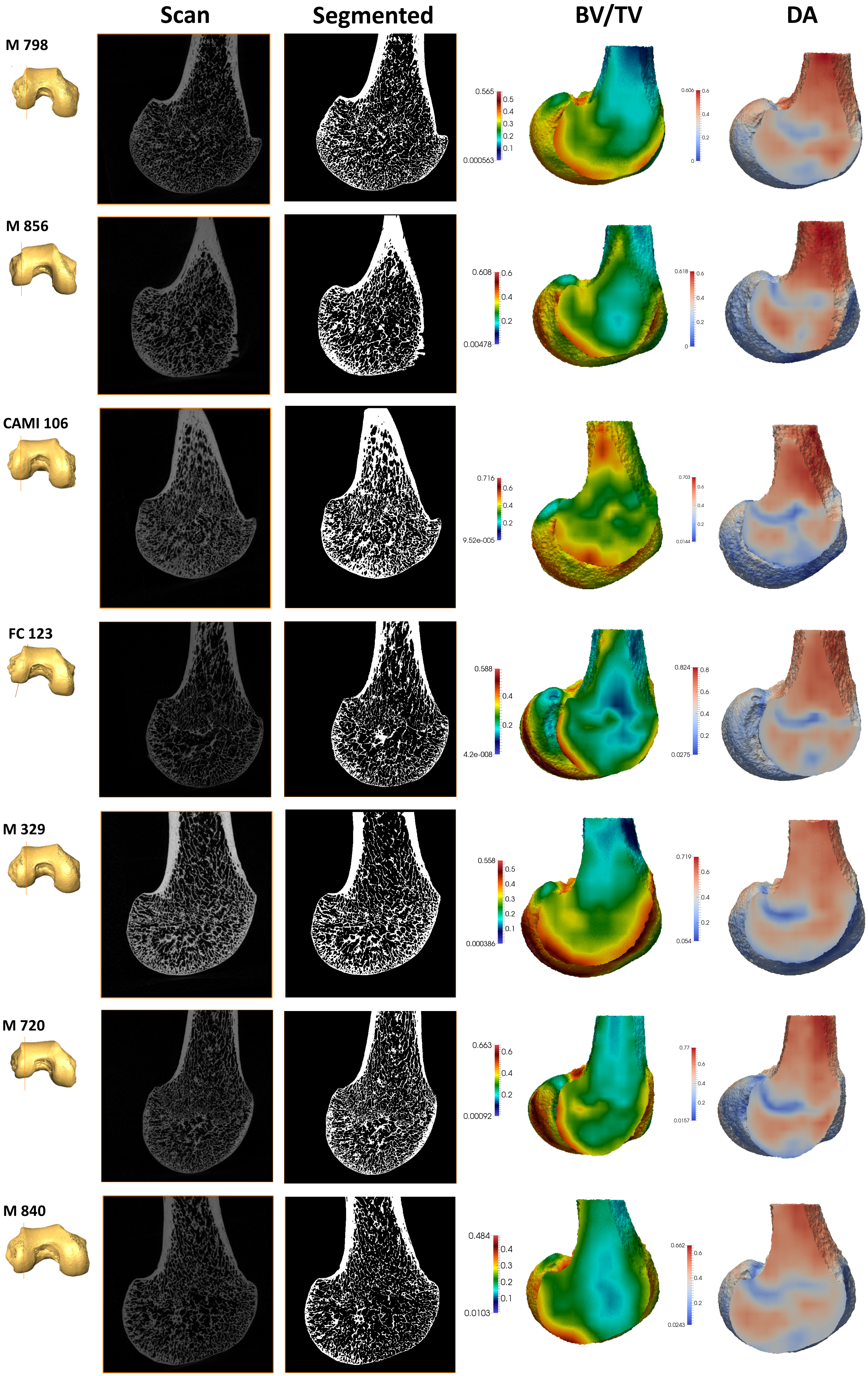

Gorilla gorilla gorilla- Medial condyle

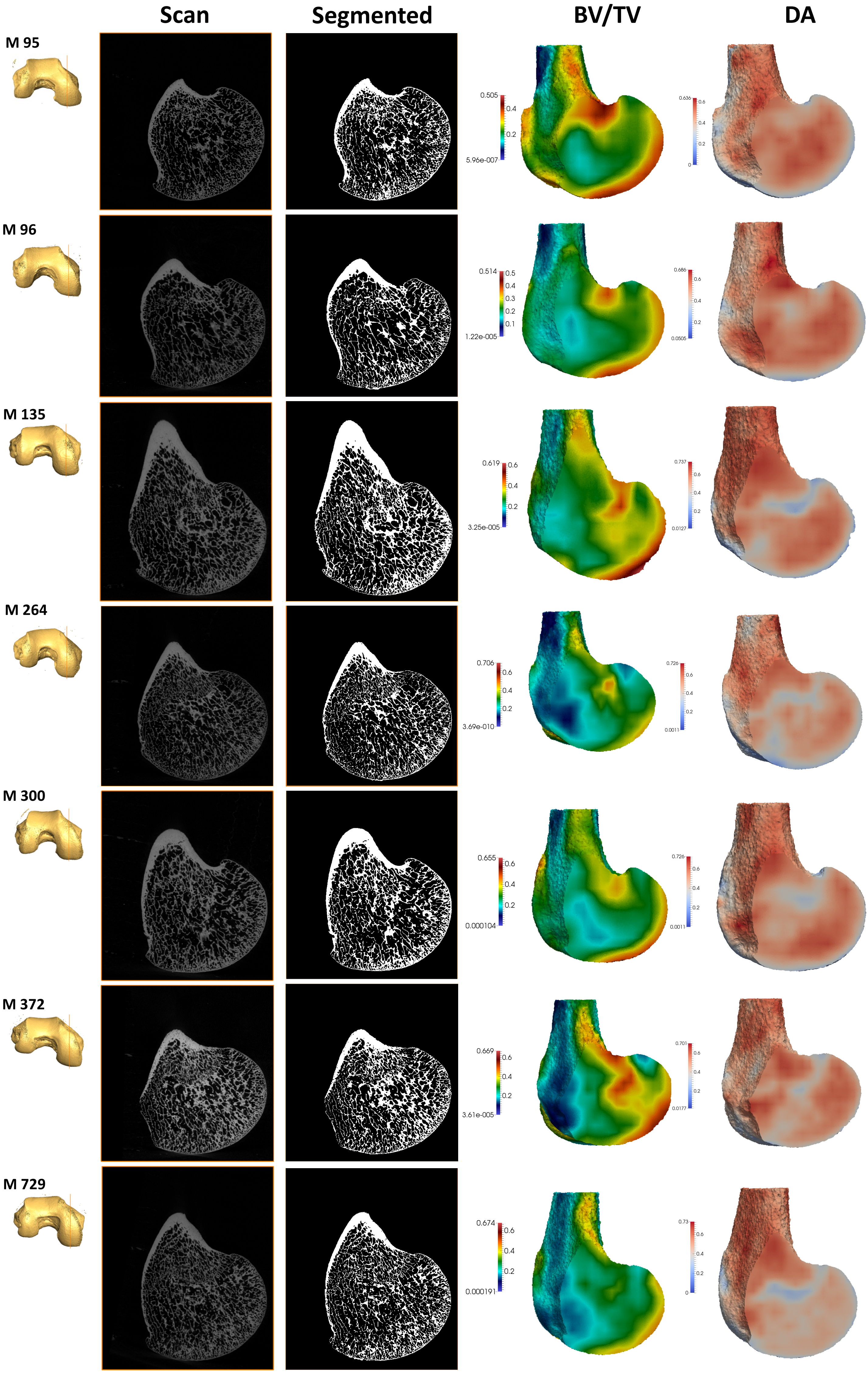

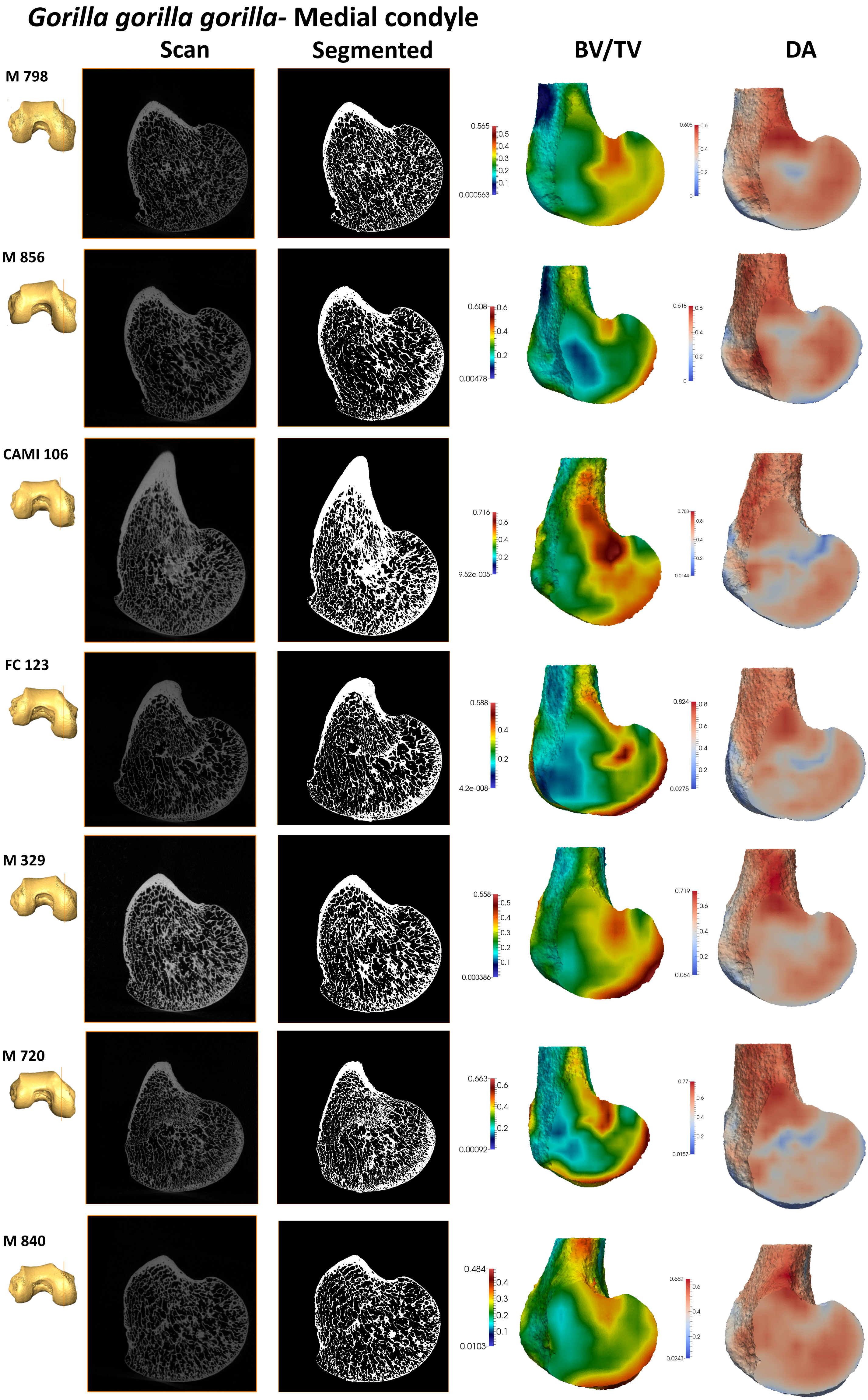

Gorilla gorilla gorilla- Medial condyle
Scan
Segmented
BV/TV
DA
M 798
M 856
CAMI 106
FC 123
M 329
M 720
M 840
