# Supplementary material for: Trabecular bone patterning in the hominoid distal femur
Source: PeerJ. 2018 Jul 5;6:e5156. doi: 10.7717/peerj.5156 (PMC6035864; doi:10.7717/peerj.5156)

Pongo sp- BV/TV distribution

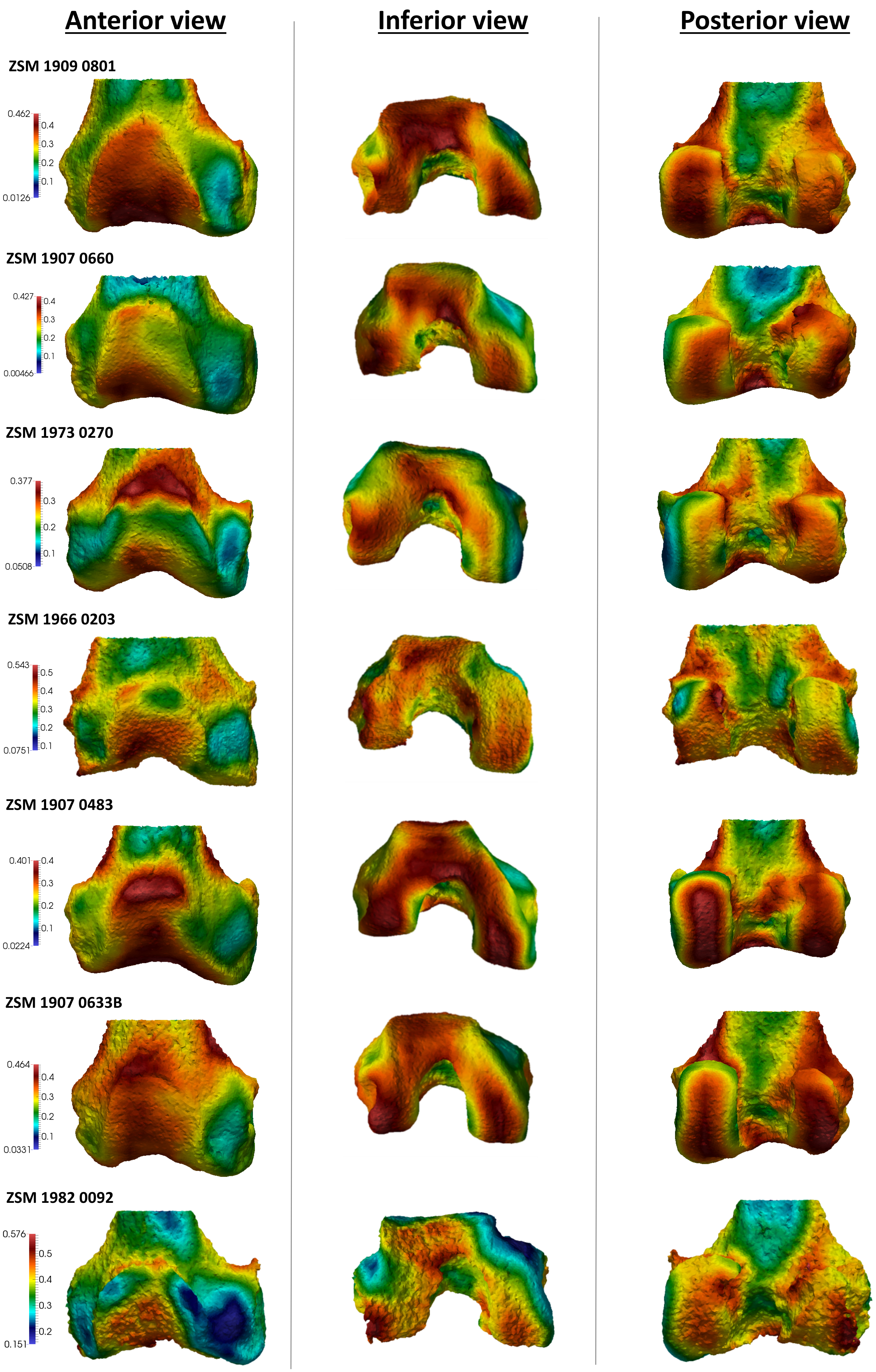

Pongo sp- Lateral condyle

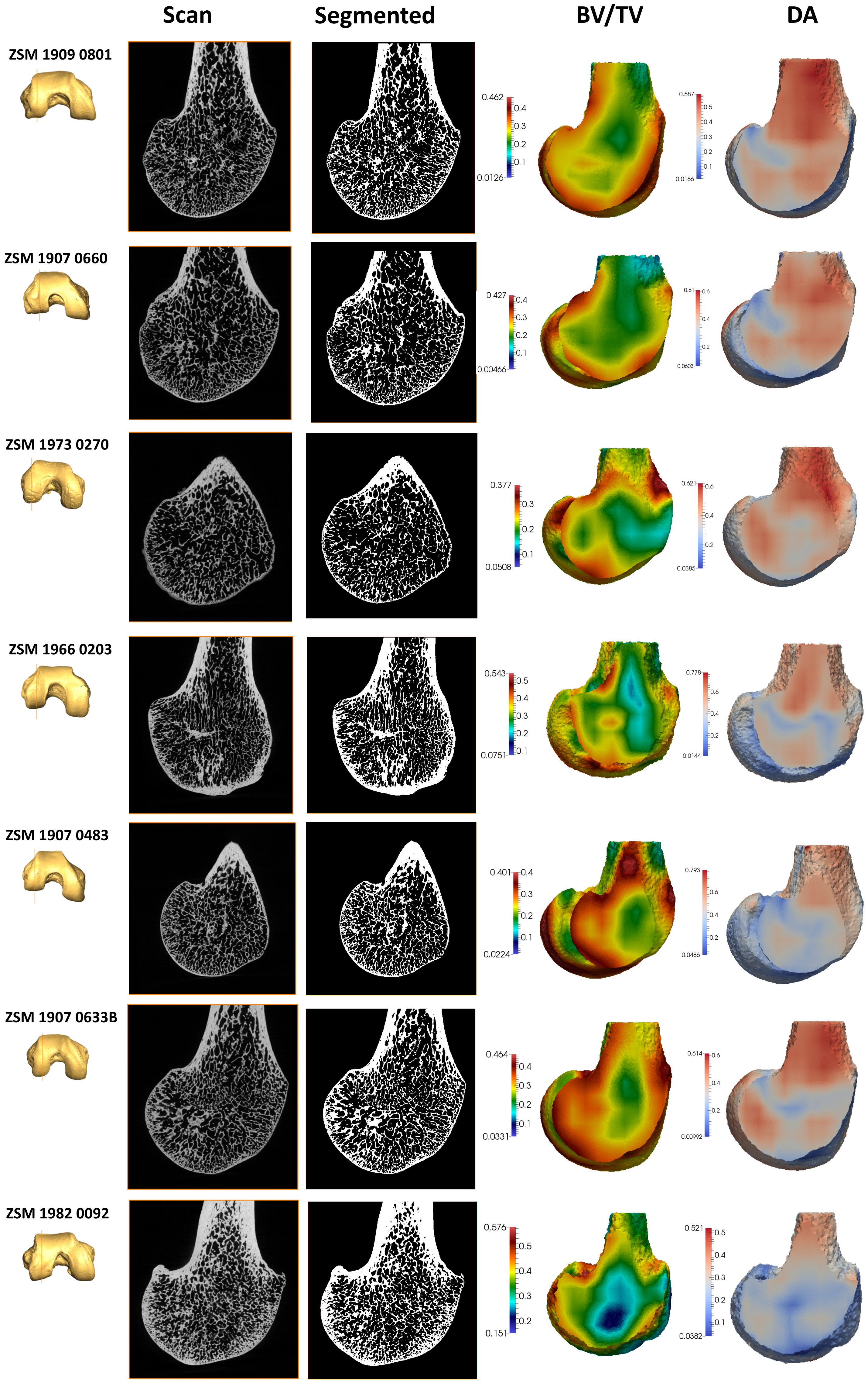

Pongo sp- Medial condyle

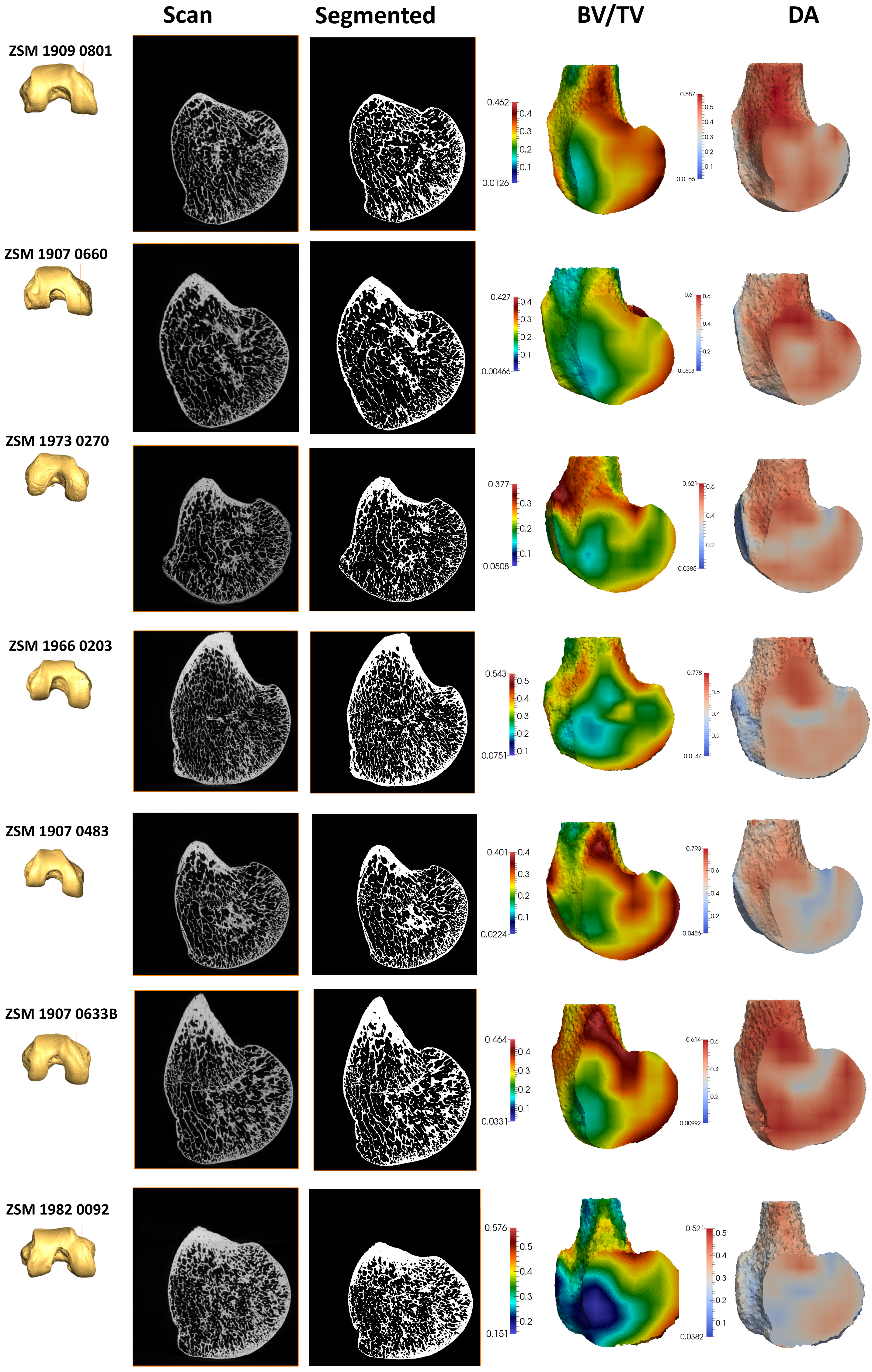

Supplement: Supplemental Information 3 — Captive specimens: ZSM 1966 0203 (male) and ZSM 1982 0092 (female). [file peerj-06-5156-s003.pdf]
