# Supplementary material for: Trabecular bone patterning in the hominoid distal femur
Source: PeerJ. 2018 Jul 5;6:e5156. doi: 10.7717/peerj.5156 (PMC6035864; doi:10.7717/peerj.5156)
Supplement: Supplemental Information 4 [file peerj-06-5156-s004.pdf]

Homo sapiens- BV/TV distribution

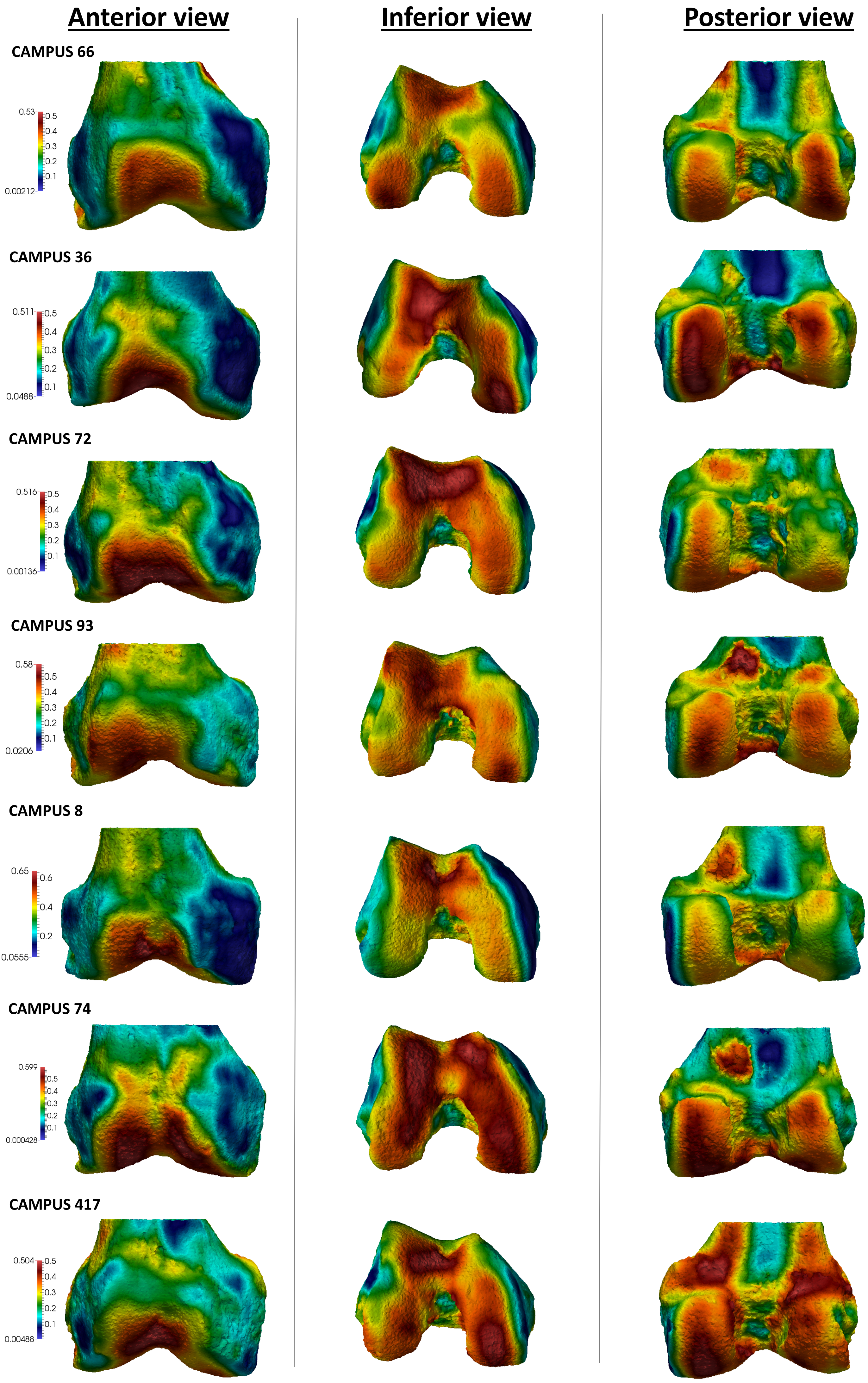

Homo sapiens- BV/TV distribution

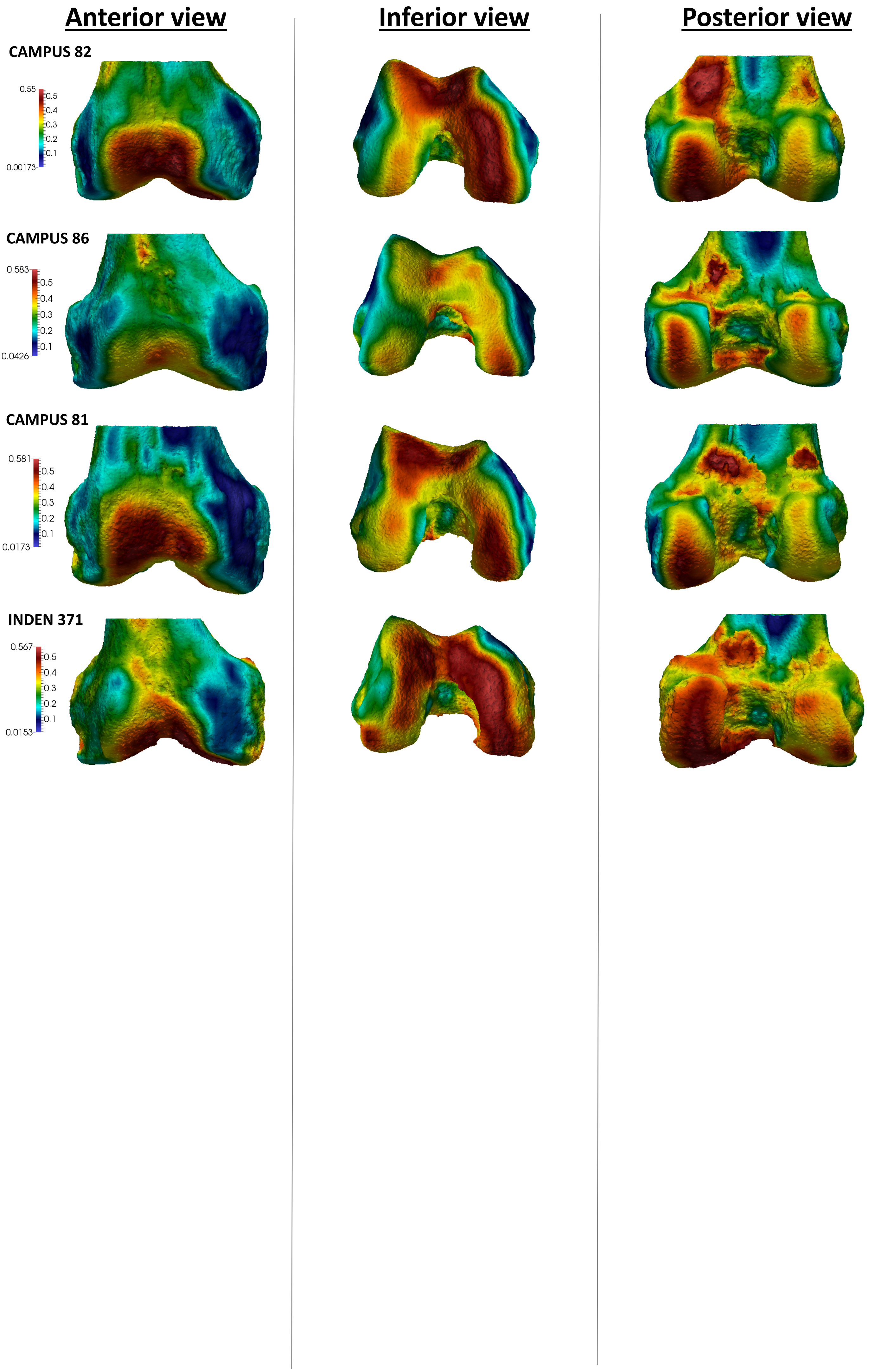

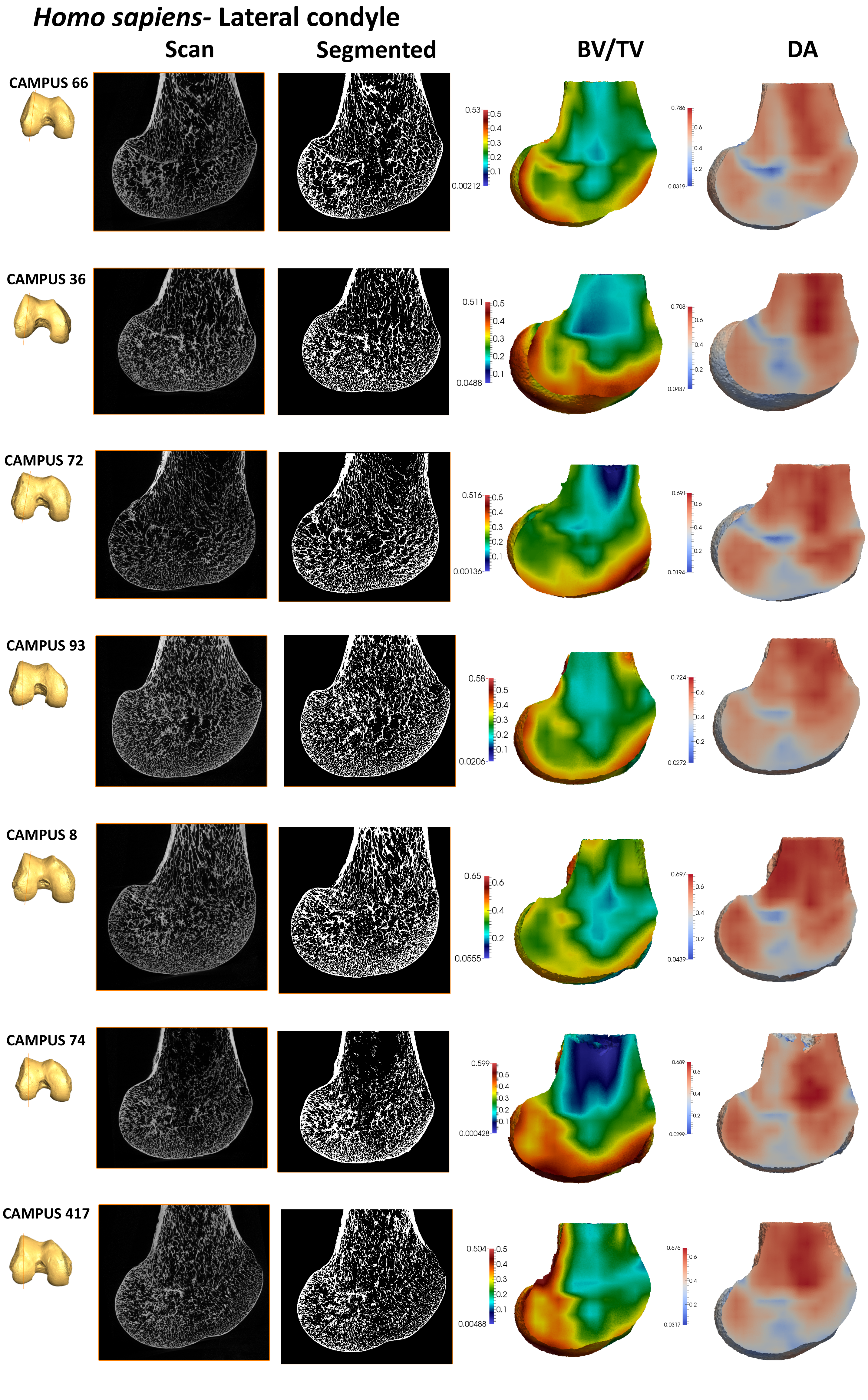
Homo sapiens- Lateral condyle
Scan
Segmented
BV/TV
DA
CAMPUS 66
CAMPUS 36
CAMPUS 72
CAMPUS 93
CAMPUS 8
CAMPUS 74
CAMPUS 417
0.53
0.00212
0.786
0.0319
0.511
0.0488
0.708
0.0437
0.516
0.00136
0.691
0.0194
0.58
0.0206
0.724
0.0272
0.65
0.0555
0.697
0.0439
0.599
0.000428
0.689
0.0299
0.504
0.00488
0.676
0.0317

Homo sapiens- Lateral condyle

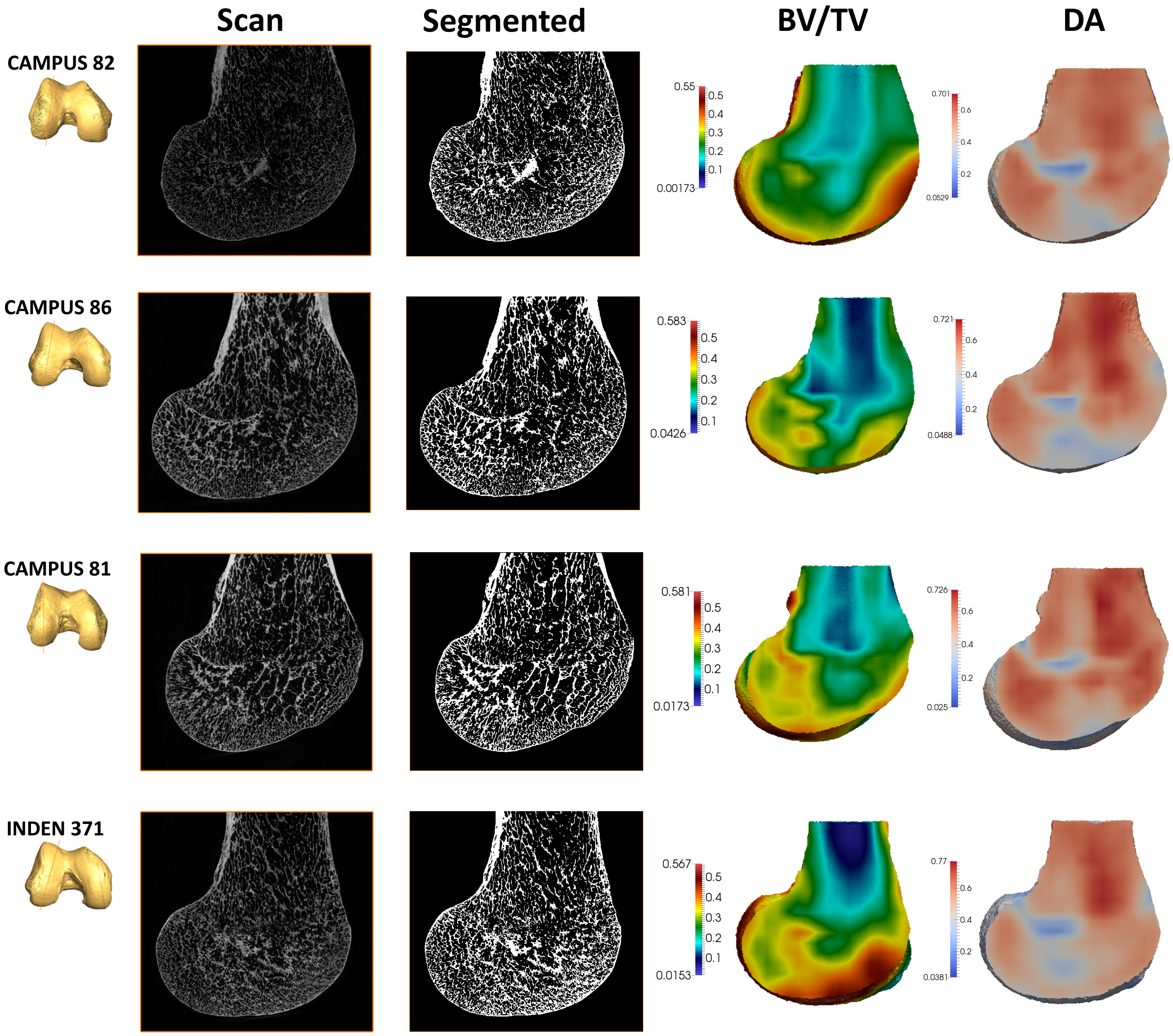

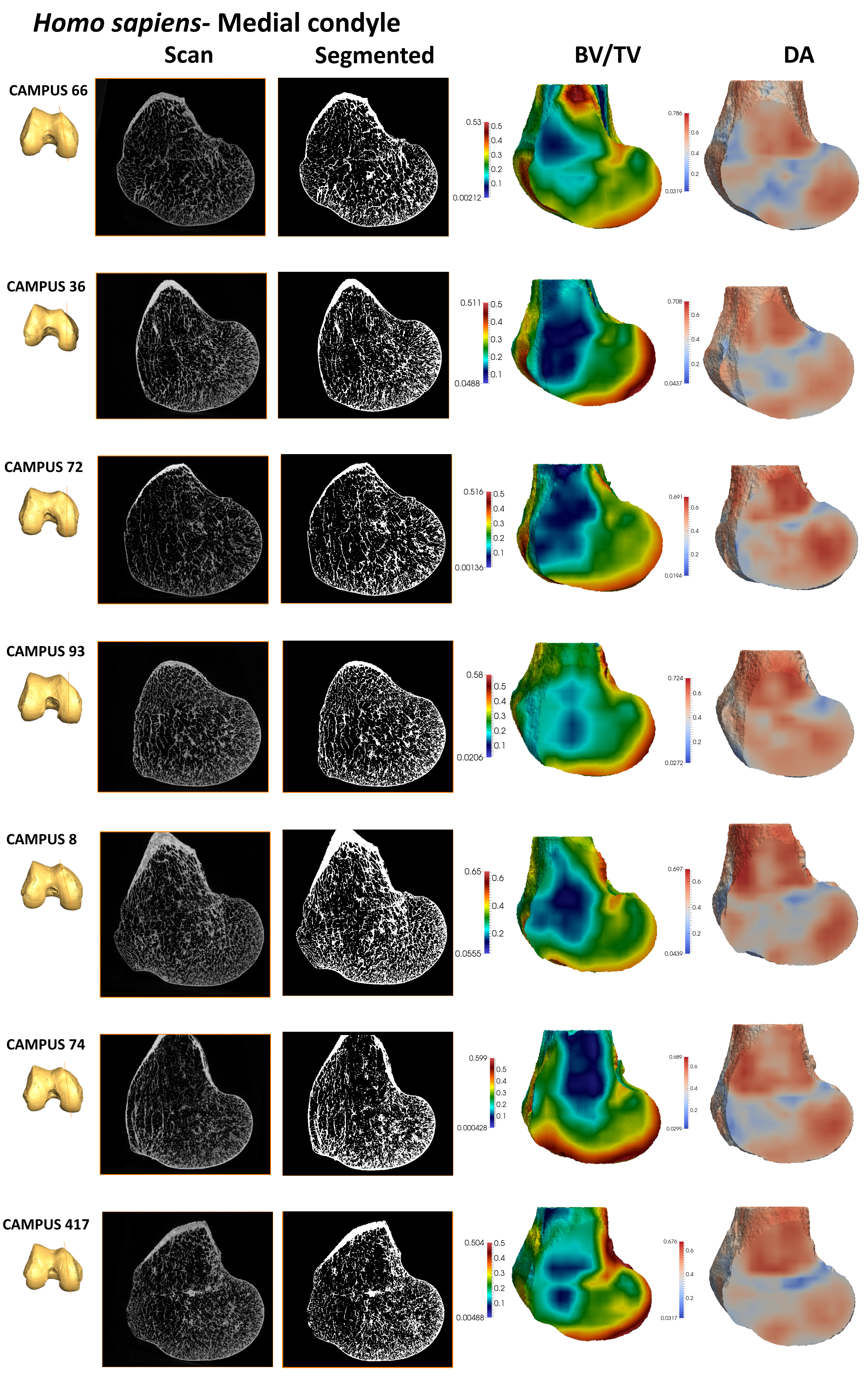

Homo sapiens- Medial condyle
Scan
Segmented
BV/TV
DA
CAMPUS 66
0.53
0.00212
0.786
0.0319
CAMPUS 36
0.511
0.0488
0.708
0.0437
CAMPUS 72
0.516
0.00136
0.691
0.0194
CAMPUS 93
0.58
0.0206
0.724
0.0272
CAMPUS 8
0.65
0.0555
0.697
0.0439
CAMPUS 74
0.599
0.000428
0.689
0.0299
CAMPUS 417
0.504
0.00488
0.676
0.0317

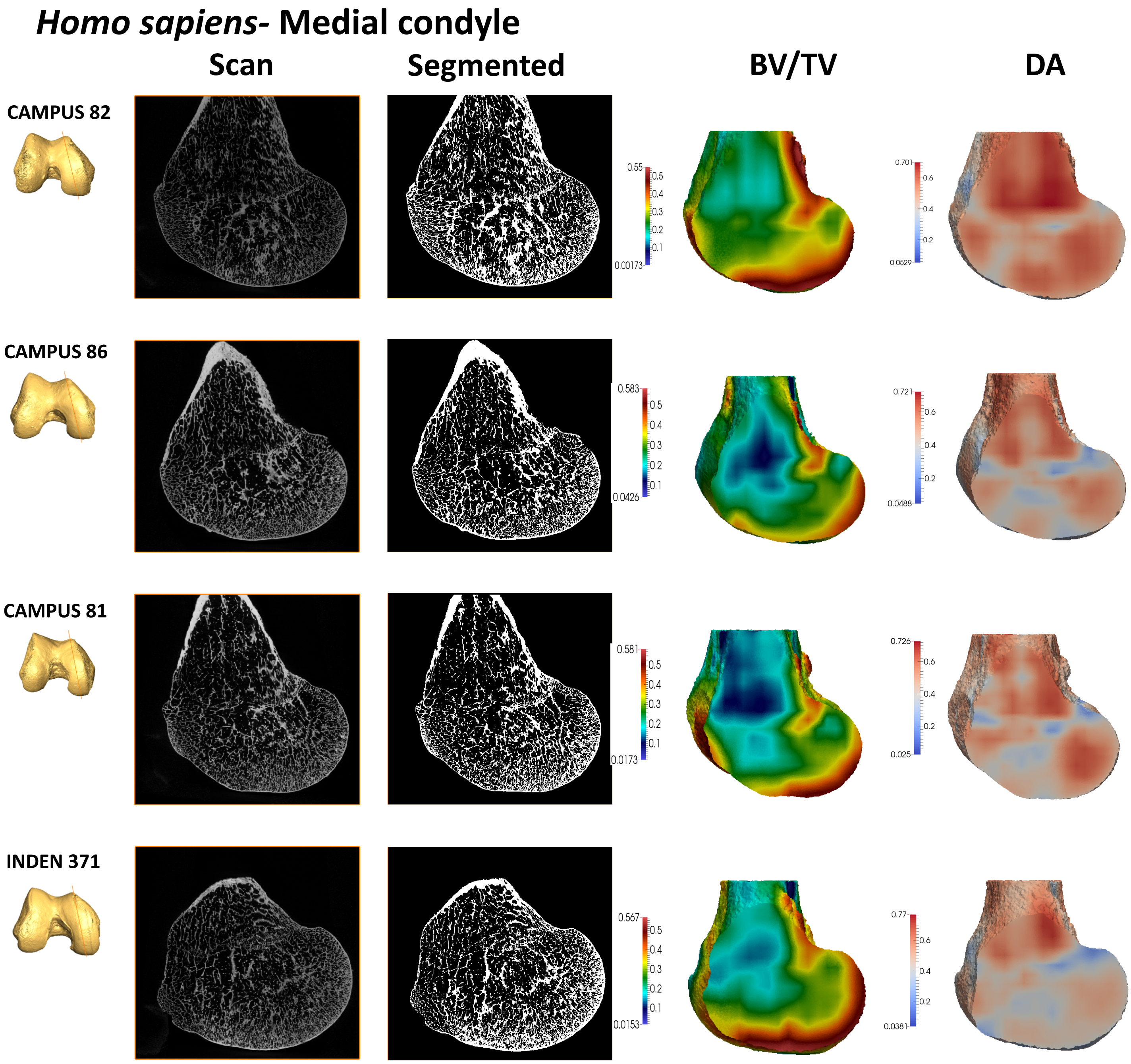
Homo sapiens- Medial condyle
Scan
Segmented
BV/TV
DA
CAMPUS 82
0.55
0.00173
0.701
0.0529
CAMPUS 86
0.583
0.0426
0.721
0.0488
CAMPUS 81
0.581
0.0173
0.726
0.025
INDEN 371
0.567
0.0153
0.77
0.0381
