# Supplementary material for: Trabecular bone patterning in the hominoid distal femur
Source: PeerJ. 2018 Jul 5;6:e5156. doi: 10.7717/peerj.5156 (PMC6035864; doi:10.7717/peerj.5156)
Supplement: Supplemental Information 5 — Captive Pongo are included. [file peerj-06-5156-s005.docx]

| Taxa | Parameter | Lateral distal | Lateral posteroinferior | Lateral posterosuperior | Medial distal | Medial posteroinferior | Medial posterosuperior |
| --- | --- | --- | --- | --- | --- | --- | --- |
| *Pan-Pongo* | BV/TV | N/A | N/A | N/A | N/A | N/A | N/A |
|  | DA | N/A | N/A | N/A | N/A | N/A | N/A |
|  | Tb.N (1/mm) | N/A | N/A | 0.02777 | N/A | N/A | 0.00430 |
|  | Tb.Sp (mm) | N/A | N/A | N/A | N/A | N/A | 0.01172 |
|  | Tb.Th (mm) | N/A | N/A | N/A | N/A | N/A | N/A |
| *Pan-Gorilla* | BV/TV | N/A | N/A | 0.03118 | N/A | N/A | 0.00900 |
|  | DA | N/A | N/A | N/A | N/A | N/A | N/A |
|  | Tb.N (1/mm) | 0.00013 | 0.00013 | 0.00013 | 0.00026 | 0.00007 | 0.00007 |
|  | Tb.Sp (mm) | 0.00007 | 0.00013 | 0.00007 | 0.00026 | 0.00007 | 0.00007 |
|  | Tb.Th (mm) | 0.00120 | 0.00190 | 0.00078 | 0.00195 | 0.00078 | 0.00007 |
| *Pan-Homo* | BV/TV | N/A | N/A | 0.00026 | N/A | N/A | 0.01700 |
|  | DA | 0.00078 | 0.00013 | 0.00013 | N/A | N/A | N/A |
|  | Tb.N (1/mm) | 0.01254 | N/A | 0.00078 | 0.00195 | 0.09000 | 0.00007 |
|  | Tb.Sp (mm) | N/A | N/A | 0.00013 | 0.00195 | N/A | 0.00013 |
|  | Tb.Th (mm) | 0.01250 | N/A | 0.03118 | 0.00078 | 0.04105 | 0.00630 |
| *Gorilla-Pongo* | BV/TV | N/A | N/A | N/A | N/A | N/A | N/A |
|  | DA | N/A | N/A | N/A | N/A | N/A | N/A |
|  | Tb.N (1/mm) | 0.00123 | 0.00432 | 0.01851 | 0.00247 | 0.00120 | 0.00120 |
|  | Tb.Sp (mm) | 0.00250 | 0.00432 | 0.01851 | 0.00432 | 0.00430 | 0.00432 |
|  | Tb.Th (mm) | 0.01170 | 0.01850 | N/A | 0.01172 | N/A | 0.04070 |
| *Gorilla-Homo* | BV/TV | N/A | N/A | N/A | N/A | N/A | N/A |
|  | DA | N/A | 0.00007 | 0.00195 | N/A | 0.01300 | N/A |
|  | Tb.N (1/mm) | 0.00435 | 0.00435 | N/A | N/A | 0.00440 | N/A |
|  | Tb.Sp (mm) | 0.00290 | 0.00195 | N/A | N/A | 0.00630 | N/A |
|  | Tb.Th (mm) | N/A | 0.04100 | N/A | N/A | N/A | N/A |
| *Pongo-Homo* | BV/TV | N/A | N/A | N/A | N/A | N/A | N/A |
|  | DA | N/A | 0.00062 | 0.00432 | N/A | 0.02800 | N/A |
|  | Tb.N (1/mm) | N/A | N/A | N/A | 0.01851 | N/A | N/A |
|  | Tb.Sp (mm) | N/A | N/A | N/A | N/A | N/A | N/A |
|  | Tb.Th (mm) | 0.04070 | N/A | N/A | 0.00740 | N/A | N/A |
