# Supplementary material for: Trabecular bone patterning in the hominoid distal femur
Source: PeerJ. 2018 Jul 5;6:e5156. doi: 10.7717/peerj.5156 (PMC6035864; doi:10.7717/peerj.5156)
Supplement: Supplemental Information 6 [file peerj-06-5156-s006.docx]

| Parameter, region | PC1 | PC2 |
| --- | --- | --- |
| Tb_Sp, lateral distal | 16.359 | 0.877 |
| Tb_Sp, lateral posteroinferior | 8.388 | 1.494 |
| Tb_Sp, lateral posterosuperior | 12.032 | 1.449 |
| Tb_Sp, medial distal | 22.391 | 0.068 |
| Tb_Sp, medial posteroinferior | 11.886 | 1.996 |
| Tb_Sp, medial posterosuperior | 20.288 | 0.028 |
| Tb_Th, lateral distal | 1.525 | 0.236 |
| Tb_Th, lateral posteroinferior | 1.380 | 1.232 |
| Tb_Th, lateral posterosuperior | 0.956 | 0.089 |
| Tb_Th, medial distal | 1.022 | 0.558 |
| Tb_Th, medial posteroinferior | 1.018 | 0.271 |
| Tb_Th, medial posterosuperior | 1.421 | 0.201 |
| DA, lateral distal | 0.571 | 8.930 |
| DA, lateral posteroinferior | 0.152 | 24.728 |
| DA, lateral posterosuperior | 0.347 | 15.028 |
| DA, medial distal | 0.071 | 6.610 |
| DA, medial posteroinferior | 0.148 | 24.134 |
| DA, medial posterosuperior | 0.045 | 12.070 |
