# Supplementary material for: Trabecular bone patterning in the hominoid distal femur
Source: PeerJ. 2018 Jul 5;6:e5156. doi: 10.7717/peerj.5156 (PMC6035864; doi:10.7717/peerj.5156)
Supplement: Supplemental Information 7 — Captive Pongo are included. [file peerj-06-5156-s007.docx]

| Taxa | Parameter | Inferior lateral index | Posterior lateral index | Inferior medial index | Posterior medial index |
| --- | --- | --- | --- | --- | --- |
| *Pan-Pongo* | BV/TV | N/A | N/A | N/A | N/A |
|  | DA | N/A | N/A | N/A | N/A |
| *Pan-Gorilla* | BV/TV | N/A | N/A | N/A | N/A |
|  | DA | 0.00292 | N/A | N/A | N/A |
| *Pan-Homo* | BV/TV | 0.0173 | N/A | N/A | 0.0173 |
|  | DA | N/A | N/A | 0.00903 | 0.01728 |
| *Gorilla-Pongo* | BV/TV | N/A | N/A | N/A | N/A |
|  | DA | N/A | N/A | N/A | N/A |
| *Gorilla-Homo* | BV/TV | 0.0063 | N/A | N/A | N/A |
|  | DA | 0.00045 | N/A | 0.00078 | 0.00078 |
| *Pongo-Homo* | BV/TV | N/A | 0.012 | N/A | 0.0074 |
|  | DA | N/A | N/A | N/A | N/A |
